# Coordination Chemistry of Mixed-Donor Pyridine-Containing Macrocyclic Ligands: From Optical to Redox Chemosensors for Heavy Metal Ions

**DOI:** 10.3390/molecules30010130

**Published:** 2024-12-31

**Authors:** Alessandra Garau, Alexander J. Blake, Maria Carla Aragoni, Massimiliano Arca, Claudia Caltagirone, Francesco Demartin, Vito Lippolis, Giacomo Picci, Enrico Podda

**Affiliations:** 1 Dipartimento di Scienze Chimiche e Geologiche, Università degli Studi di Cagliari, S.S. 554 Bivio per Sestu, 09042 Monserrato, Italy; 2 School of Chemistry, University of Nottingham, University Park, Nottingham NG7 2RD, UK; 3 Dipartimento di Chimica, Università degli Studi di Milano, Via Golgi 19, 20133 Milano, Italy; 4 Centre for Research University Services (CeSAR), Università degli Studi di Cagliari, S.S. 554 Bivio per Sestu, 09042 Monserrato, Italy

**Keywords:** macrocyclic ligand, ferrocene, redox chemosensor, lead, heavy metal ions

## Abstract

2,8-Dithia-5-aza-2,6-pyridinophane (**L1**) has been used as a receptor unit in the construction of the conjugated redox chemosensor 5-ferrocenylmethyl-2,8-dithia-5-aza-2,6-pyridinophane (**L3**). In order to further explore the coordination chemistry of **L1**, and comparatively, that of its structural analogue 2,11-dithia-5,8-diaza-2,6-pyridinophane (**L2**), featuring two secondary nitrogen atoms in the macrocyclic unit, the crystal structures of the new synthesised complexes [Pb(**L1**)(ClO_4_)_2_]·½CH_3_CN, [Cu(**L2**)](ClO_4_)_2_·CH_3_CN and [Cd(**L2**)(NO_3_)]NO_3_ were determined by X-ray diffraction analysis. The electrochemical response of **L3** towards the metal ions Cu^2+^, Zn^2+^, Cd^2+^, Hg^2+^, and Pb^2+^ was investigated by cyclic voltammetry (CV) in CH_2_Cl_2_/CH_3_CN 0.25:1 (*v*/*v*) mixture. Upon addition to **L3** of increasing amounts of the aforementioned metal cations, the wave corresponding to the Fc^+^/Fc redox couple of the un-complexed **L3** was gradually replaced by a new reversible wave at more positive potentials and corresponding to the Fc^+^/Fc redox couple of the complexed ligand. The maximum anodic shift of the ferrocene oxidation wave is observed in the presence of Pb^2+^ (230 mV), to which corresponds a reaction coupling efficiency (RCE) value as large as 7.9 × 10^3^. The response selectivity of **L3** is discussed in reference to the optical selectivity observed for conjugated chemosensors featuring **L1** as receptor unit and different fluorogenic fragments as signalling units.

## 1. Introduction

The development of sensitive and selective analytical tools for the rapid detection and monitoring of toxic heavy metal ions (such as Hg^2+^, Cd^2+^, and Pb^2+^) in environmental, industrial, and biological samples is a very active area of research [1,2,3,4,5]. These cations can be seriously deleterious to human health, and their use in many industrial processes results in a highly concerning level of contamination in soil, water and food, thus representing a huge pollution problem [6,7,8,9,10,11,12].

The development of selective and sensitive molecular sensors or chemosensors de- signed according to the principles of supramolecular chemistry [13,14,15,16,17,18,19,20,21,22] and the related sensor technology (sensor arrays, electro/optical noses and tongues) [23,24,25,26,27] represent well-established possible solutions to this difficult analytical problem due to their simplicity, versatility, low response time and cost.

From a structural point of view, according to the supramolecular “receptor-spacer-active unit” paradigm, conjugated chemosensors featuring a signalling site (active unit) linked through an appropriate spacer to a binding site (receptor unit) are the most studied and exploited. The recognition event, namely the host–guest interaction of the target species with the receptor unit, is converted into detectable changes in the chemical/physical properties of the active unit. Such changes can be exploited for analytical purposes provided that the chemosensor shows a signalling selectivity for the target species.

Over the last decade, by applying this general supramolecular synthetic approach, a great number of optical/electrochemical-conjugated chemosensors based on polyoxa- polyaza-, and aza/oxa-macrocycles as the guest binding sites have been reported. This choice can guarantee good water solubility, synthetic accessibility and binding selectivity towards hard and borderline hard/soft metal ions following an easily achievable ring size complementarity with the guest dimensions [28,29,30,31]. On the other hand, the potential of S- or mixed N/S-, and N/S/O-macrocyclic binding sites has in comparison been barely explored, reflecting a generally increased difficulty encountered both in the synthetic preparation of these systems [32,33,34,35,36,37,38], and in achieving a high binding selectivity towards target analytes within the category of soft metal ions with marked thiophilicity [28,29,30,31]. Fortunately, signalling selectivity can still be achieved with chemosensors featuring sulphur-containing donor atoms, and is dependent on the choice of the signalling unit and the medium for the host–guest interaction.

**Figure 1:**
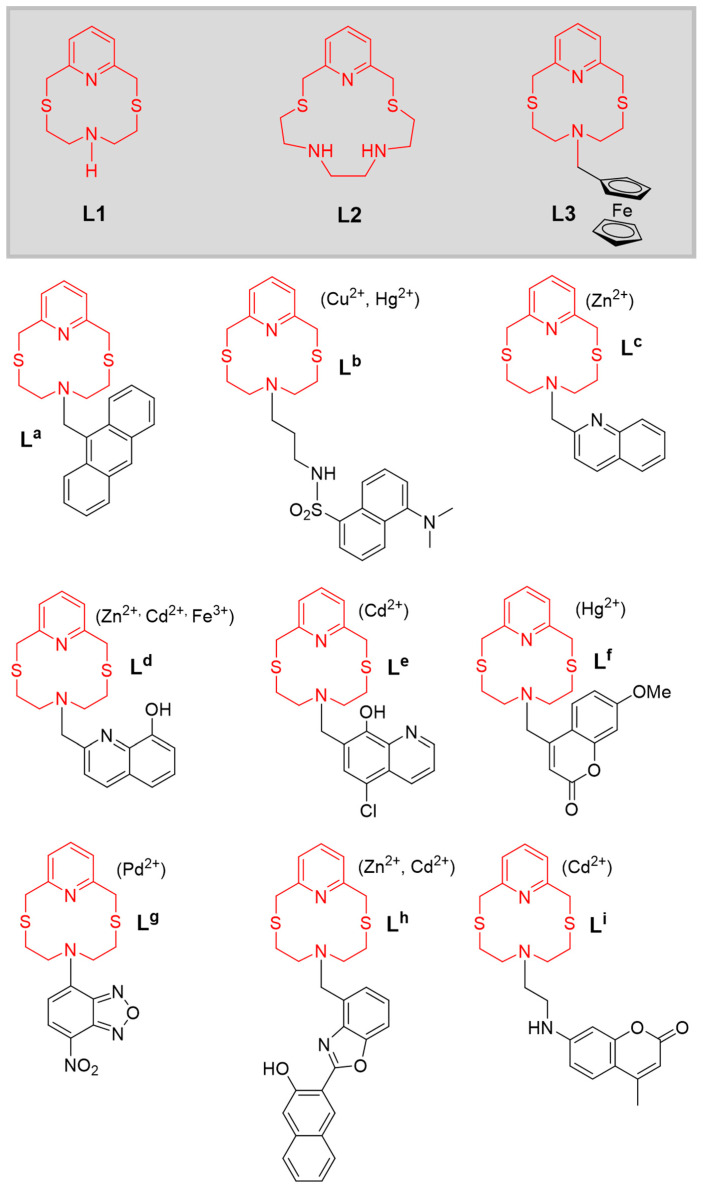
Molecular schemes of the macrocyclic ligands **L1**, **L2** and **L3**, and fluorescent chemosensors for metal ions **L^a^**–**L^i^** built using **L1** as the receptor unit (optical selectivity in parentheses) [39,40,41,42,43,44,45,46,47].

Following our general interest in the coordination chemistry of macrocyclic ligands aimed at application purposes, we have developed numerous optical chemosensors featuring the 12-membered pyridine-based macrocycle 2,8-dithia-5-aza-2,6-pyridinophane (**L1**) as a receptor unit, and various covalently linked fluorogenic fragments as a signalling unit (**L^a^**–**L^i^** in Figure 1) [39,40,41,42,43,44,45,46,47]. A different optical selectivity (indicated in parentheses in Figure 1) was observed for **L1**-based developed chemosensors, depending on the nature of the signalling unit and the experimental conditions used for the host–guest interaction. The observed optical selectivity does not generally correspond to the thermodynamic stabilities of the 1:1 complexes formed by **L1** with the same metal ions, but this did not preclude the use of the chemosensors in the analytical determination of the target metal ions in real matrices of environmental and biological provenance. In fact, despite **L1** not exhibiting a marked binding selectivity for any of the metal ions among Cu^2+^, Zn^2+^, Cd^2+^, Hg^2+^, and Pb^2+^, its functionalisation with different fluorogenic fragments has allowed the construction of optically selective fluorescent chemosensors as outlined in Figure 1 [39,40,41,42,43,44,45,46,47]. In some cases, a binding selectivity was achieved by supporting the resulting chemosensors in PVC membranes or nanoparticles where other parameters (e.g., the lipophilicity of the chemosensors and their metal complexes, their mobility within the membrane, and other factors determining the transport processes) can improve the binding selectivity and the sensitivity of the chemosensor observed in solution (for a detailed analysis and discussion of the optical properties in the presence of metal cations and applications of the chemosensors **L^a^**–**L^i^**, refer to the relevant references [39,40,41,42,43,44,45,46,47]).

The synthetic “receptor-spacer-active unit” modular scheme outlined above does not necessarily apply to the construction of chemosensors involving an optical transduction mechanism for the host–guest interaction, so linking a redox-active centre to a receptor unit may allow a selective electrochemical detection of binding via a redox potential change of the reporter group (voltammetric sensing). In this context, ferrocene has been widely studied in the field of chemosensing, especially in combination with macrocyclic receptor units, due to its well-defined electrochemical profile, chemical stability and ease of functionalisation on either one or both of its cyclopentadienyl (Cp) rings [48,49,50,51,52].

In this paper, we provide new insight into the coordination chemistry of **L1** towards the dipositive metal ions Cu^2+^, Zn^2+^, Cd^2+^, Hg^2+^, and Pb^2+^. We also compare the behaviour of **L1** with that of its structural analogue 2,11-dithia-5,8-diaza-2,6-pyridinophane (**L2**, Figure 1), featuring two secondary nitrogen atoms in the macrocyclic cavity. The synthesis of the *N*-ferrocenylmethyl derivative **L3** (Figure 1) was also achieved, and its electrochemical response to the above-mentioned metal ions was also investigated by cyclic voltammetry (CV), underlining the synergic cooperation between the receptor and signalling units within conjugated chemosensors in achieving signalling selectivity.

## 2. Results

### 2.1. Coordination Chemistry of ***L1*** and ***L2***

We have previously observed that the 1:1 complex cations [M(**L1**)]^2+^, (M = Cu^2+^, Zn^2+^, Cd^2+^, Hg^2+^, and Pb^2+^) are formed in aqueous solutions with formation constants (log *K*, determined potentiometrically) increasing in the order Zn^2+^ (7.13) < Pb^2+^ (8.45) < Cd^2+^ (9.12) < Cu^2+^ (10.05) < Hg^2+^ (10.68) [39]. A similar trend [Cd^2+^ (5.6) < Zn^2+^ (9.56) < Pb^2+^ (11.01) < Cu^2+^ (14.70) < Hg^2+^ (14.95)] was observed in CH_3_CN/H_2_O (1:1 *v*/*v*) solution for **L2** (Figure 1), the structural analogue of **L1** featuring a larger ring size due to the presence of two secondary amine groups in its structure [41]. As expected, the binding constants measured for **L2** are generally higher than those measured for **L1** due to the presence of five donor atoms and the larger cavity in the former. Interestingly, despite Pb^2+^ being third in the order of binding affinity, the **L2**-based chemosensor featuring two (5-chloro-8-hydroxy-7-quinolinyl)methyl pendant arms shows higher optical selectivity towards Pb^2+^ [41] than towards Cu^2+^ and Hg^2+^; a similar behaviour is in general observed for the **L1**-based chemosensors shown in Figure 1, most of which display a high optical selectivity for Zn^2+^ and/or Cd^2+^, i.e., the metal ions featuring the lowest binding constant with **L1**.

The coordination chemistry in the solid state of both **L1** and **L2** towards Cu^2+^ and *d*^10^ metal ions such as Zn^2+^, Cd^2+^, Hg^2+^, and Pb^2+^ has barely been studied [53]: for **L1**, only the 1:1 complexes [Cu(**L1**)(NO_3_)_2_] and [Zn(**L1**)(NO_3_)_2_] were reported as nitrate salts [39], while no X-ray crystal structures of **L2**-based metal complexes are known. The coordination spheres around the metal centres in both [Cu(**L1**)(NO_3_)_2_] and [Zn(**L1**)(NO_3_)_2_] complexes are *pseudo*-octahedral, with four positions occupied by the donor atoms of the macrocyclic ligand in a folded conformation, and the remaining two mutually *cis*-positions accommodating two unidentate nitrate anions. In order to have an insight into the solid state binding properties of **L1** towards larger *d*^10^ metal ions, we reacted the ligand with Pb(ClO_4_)_2_·3H_2_O (1:1 molar ratio) in CH_3_CN. The colourless crystals obtained by diffusion of Et_2_O vapour into the reaction mixture, and corresponding to the formulation [Pb(**L1**)(ClO_4_)_2_]·½CH_3_CN were analysed by X-ray diffraction on a single crystal [see Table S1 in the Supporting Information (SI) for the crystallographic parameters].

**Figure 2:**
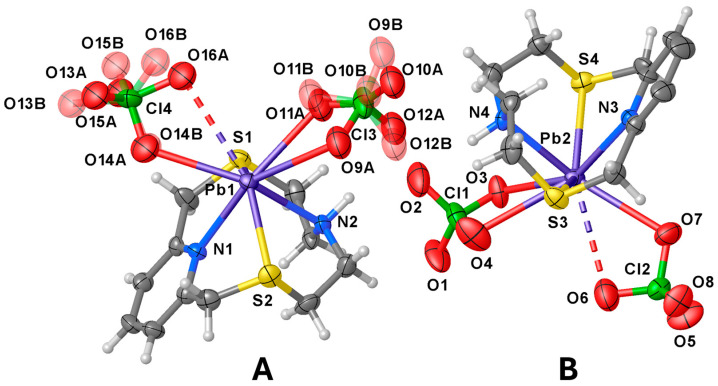
View of the two independent [Pb(**L1**)(ClO_4_)_2_] complex units (**A**,**B**) present in the asymmetric unit of compound [Pb(**L1**)(ClO_4_)_2_]·½CH_3_CN with the numbering scheme adopted. Displacement ellipsoids are drawn at 30% probability level. The co-crystallised CH_3_CN molecule is omitted for clarity. In the case of unit **A**, the disorder components of the coordinated ClO_4_^−^ anions are shown. The Pb–O contacts longer than 3.2 Å are drawn as dashed lines (see Tables S2 and S3 in the SI for a list of selected bond distances and angles).

The asymmetric unit features two independent [Pb(**L1**)(ClO_4_)_2_] complex units (**A** and **B** in Figure 2) and one CH_3_CN molecule. In both complex units the metal ion is coordinated to **L1** through all four donor atoms of the macrocyclic ligand [Pb–N and Pb–S distances in the range 2.529(6)–2.566(7) Å and 2.846(2)–2.878(2) Å, respectively], and to two perchlorate anions, one acting in a unidentate (κ^1^*O*) [Pb1–O14A = 2.89(2), Pb2–O7 = 2.999(7) Å] and the other in a chelating bidentate (κ^2^*O*,*O*′) fashion to afford an overall hepta-coordination [Pb1–O9A = 2.954(15), Pb1–O11A = 2.78(3) Å; Pb2–O3 = 2.822(7), Pb2–O4 = 3.059(11) Å, Figure 2]. It is worth noting that although O16A and O6 point towards the metal centres they sit at significantly long distances from Pb1 and Pb2, respectively [Pb1–O16A = 3.294(18), Pb2–O6 = 3.339(11) Å] (see discussion below).

The macrocycle in each independent [Pb(**L1**)(ClO_4_)_2_] unit assumes the folded conformation already observed in both [Cu(**L1**)(NO_3_)_2_] and [Zn(**L1**)(NO_3_)_2_] [28]. Interestingly, as compared to the Cu^2+^ and Zn^2+^ complexes, in [Pb(**L1**)(ClO_4_)_2_] the N–Pb–N and S–Pb–S angles {83.5(2) [unit **A**], 82.5(2) [unit **B**], and 130.12(8) [unit **A**], 129.97(8)^o^ [unit **B**], respectively} are significantly smaller than those observed in the Cu^2+^ and Zn^2+^ complexes of **L1** [N–M–N = 90.47(1) and 90.48(12)^o^ for M = Cu and Zn, respectively; S–M–S = 165.78(7) and 163.50(4)^o^ for M = Cu and Zn, respectively] [28], indicating a displacement of the Pb^2+^ ion out of the ring cavity towards the O-donor manifold of the perchlorate anions. This structural aspect of the coordination chemistry of **L1**, presumably determined by the larger ionic radius of Pb^2+^ with respect to that of Cu^2+^ and Zn^2+^ and/or by the presence of a stereochemically active 6*s*^2^ lone pair for lead(II), is also observed upon comparing the same bond angles in the compounds [Cu(**L^c^**)](ClO_4_)_2_·½CH_3_CN, [Zn(**L^c^**)(H_2_O)](ClO_4_)_2_, [Hg(**L^c^**)(CH_3_CN)](ClO_4_)_2_, and [Pb(**L^c^**)(ClO_4_)_2_] (Figure 1). In these four complexes, the ligand coordinates the metal centres via all five donor atoms including the N-donor from the quinoline moiety, the coordination sphere being completed by a water molecule and an acetonitrile molecule in the case of the zinc(II) and mercury(II) complexes, respectively, and two perchlorate anions in the case of the lead(II) one [40]. The Pb–O(ClO_4_^−^) bond distances with the perchlorate anions fall in the range 2.70–3.10 Å, which comprises 65% of the Pb–O(ClO_4_^−^) distances retrieved from the Cambridge Structural Database (CSD); 72% of all reported Pb–O(ClO_4_^−^) distances fall in the range 2.70–3.20 Å (CSD version 5.45 accessed on 20 August 2024, see Figure S1 in the SI and refs. [54,55,56,57,58] for some examples of discrete binuclear lead(II) complexes featuring perchlorate anions bridging the two metal ions). However, except for Pb1–O14A and Pb1–O11A in asymmetric unit **A**, and Pb2–O3 in unit **B**, the other Pb–O distances (see Figure 2) should be rather considered as contacts due to their length exceeding the sum of the Shannon ionic radii of lead(II) (1.23, 1.29 and 1.35 Å for hepta-, octa-, and nona-coordinated metal ion, respectively) [59] and the van der Waals radius of oxygen (1.50 Å) [60].

**Figure 3:**
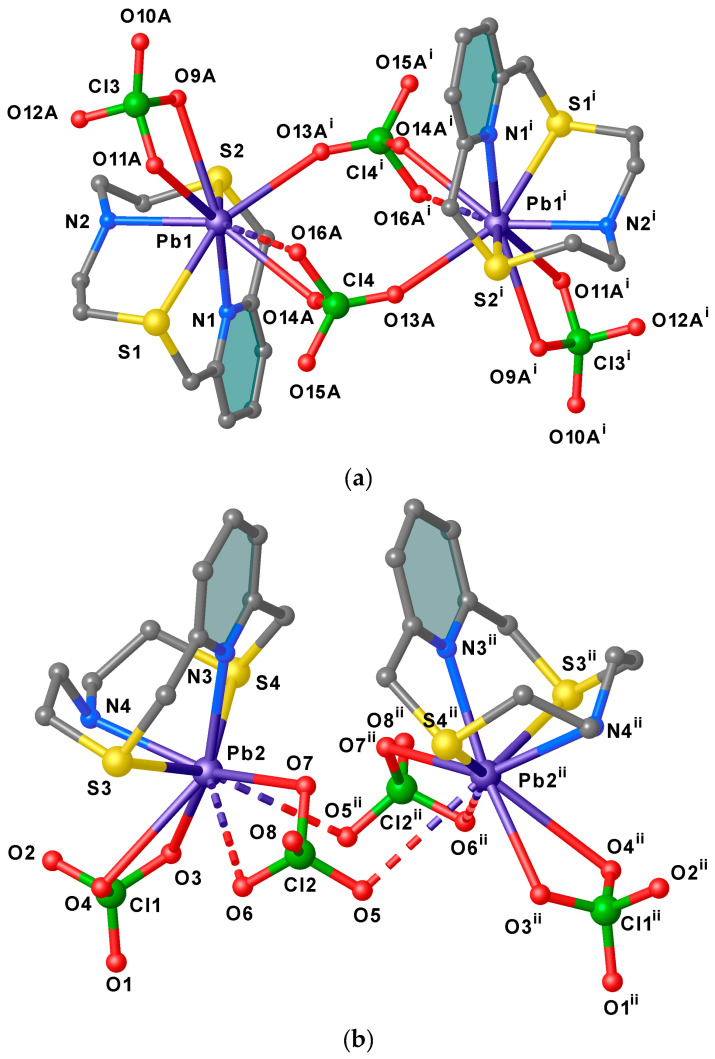
View of the dimeric arrangements of units **A** (**a**) and **B** (**b**) in [Pb(**L1**)(ClO_4_)_2_]·½CH_3_CN with the adopted numbering scheme. Hydrogen atoms are omitted for clarity. The Pb–O contacts longer than 3.2 Å are drawn as dashed lines. Symmetry codes: ^i^ **=** 1 − x, 1 − y, 1 − z; ^ii^ = 2 − x, +y, 3/2 − z.

Two asymmetric **A** units are bridged by a μ^2^- κ^1^O, κ^1^O′ perchlorate ion through O13A and O14 atoms [Pb1–O13A^i^ = 2.83(3), Pb1–O14A 2.89(2) Å, ^i^ = 1 − x, 1 − y, 1 − z] to form dimers lying across an inversion centre (Figure 3a), and each metal centre reaching an overall octa-coordination. In these dimers the two pyridyl rings assume an anti-periplanar arrangement with respect the Pb1−Pb1^i^ axis (Figure 3a). Similarly, two **B** units related by a two-fold rotation axis are arranged in pseudo-dimers with the pyridyl rings in a periplanar disposition with respect the Pb2–Pb2^ii^ axis (^ii^ = 2 − x, +y, 3/2 − z) (Figure 3b). However, considering the quite long Pb2−O5^ii^ distance [3.296(8) Å, only 4% of all reported Pb–O(ClO_4_*^−^*) distances in the CSD occupy the range 3.20–3.40 Å (see SI, Figure S1 and Table S3] [61,62,63,64,65,66], and despite the fact that O5 points towards the Pb2^ii^, the two symmetry-related **B** units cannot be considered as bridged by perchlorate anions as for the case of the dimeric aggregates formed by unit **A**. This structural feature might be indicative of the presence of a stereochemically active 6s^2^ lone pair positioned in the coordination hemisphere left free by the macrocyclic ligand for the lead(II) atom in unit **B**. Interestingly, among the 26 structures (out of a total of 106) reported in the CSD of lead(II) complexes featuring bridging perchlorate anions [Pb_2_(μ_2_−κ^1^O,κ^1^O′−ClO_4_)], very few are characterised by short Pb–O(ClO_4_*^−^*) bridging bond lengths similar to those observed in the dimers formed by the asymmetric units **A** in [Pb_2_(**L1**)_2_(ClO_4_)_4_]·½CH_3_CN (Figure 3a) [67,68,69].

**Figure 4:**
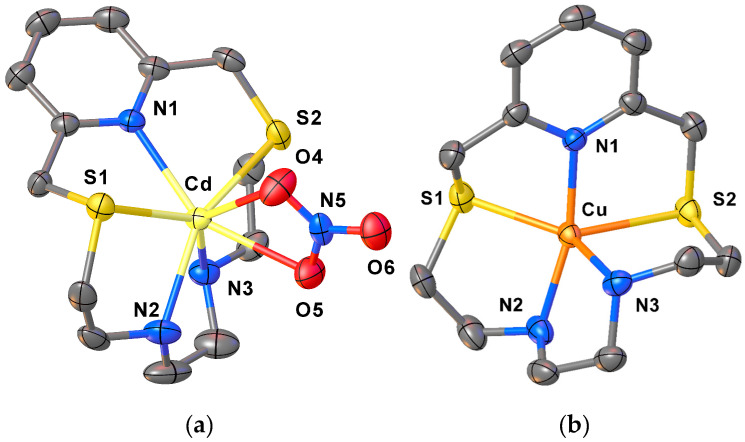
View of the complex cations [Cd(**L2**)(NO_3_)]^+^ (**a**), and [Cu(**L2**)]^2+^ (**b**) in [Cd(**L2**)(NO_3_)]NO_3_ and [Cu(**L2**)](ClO_4_)_2_·CH_3_CN, respectively, with the numbering scheme adopted. Displacement ellipsoids are drawn at 50% probability level. Hydrogen atoms, counter anions and co-crystallised solvent molecules have been omitted for clarity (see Table S4 in the SI for a list of selected bond distances and angles).

With the aim of obtaining more information on the chelating ability of **L2** in the solid state, we treated this ligand with Cu^2+^, Zn^2+^, Cd^2+^ or Pb^2+^ as a nitrate or a perchlorate salt in 1:1 molar ratio in CH_3_CN or EtOH. Single crystals suitable for X-ray diffraction analysis were successfully grown for compounds [Cu(**L2**)](ClO_4_)_2_·CH_3_CN and [Cd(**L2**)(NO_3_)]NO_3_, while crystallisation with the other two metal ions led to uncharacterizable rubbery/oily compounds. Figure 4 shows the coordination sphere around the metal centres in the two metal complex cations [Cd(**L2**)(NO_3_)]^+^ and [Cu(**L2**)]^2+^, and Table S4 in the SI summarises selected bond distances and angles.

In [Cd(**L2**)NO_3_]^+^, an overall N_3_S_2_O_2_ hepta-coordination is achieved at the metal centre via the five donor atoms of the macrocyclic framework [Cd–N1 2.416(4), Cd–N2 2.397(4), Cd–N3 2.338(4), Cd–S1 2.7264(13), Cd–S2 2.7695(13) Å] and two O-donors from one asymmetrical bidentate NO_3_*^−^* anion [Cd–O4 2.380(4) and Cd–O5 2.516(4) Å] (Figure 4a, Table S4 in the SI).

**Figure 5:**
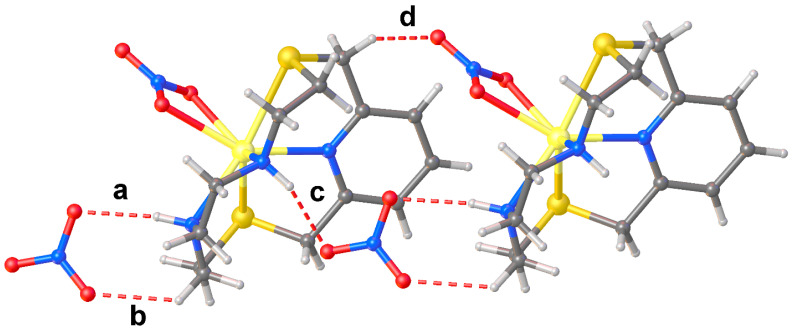
Partial view (ball and stick representation) of the extended chains of H-bonded [Cd(**L2**)NO_3_]^+^ cations and NO_3_*^−^* anions in [Cd(**L2**)NO_3_]NO_3_. Hydrogen bonds are shown as dashed lines. (**a**) N2–H2···O3 [N2–H2 (0.93 Å), N2···O3 (3.159(8) Å), H2···O3 (2.25 Å), N2–H2···O3 (167°)]; (**b**) C9–H9A···O1 [C9–H9A (0.99 Å), C9···O1 (3.031(8) Å), H9A···O1 (2.39 Å), C9–H9A···O1 (122°)]; (**c**) N3–H3···O2^i^ [N3–H3 (0.93 Å), N3···O2^i^ (2.928(6) Å), H3···O2^i^ (2.09 Å), N3–H3···O2^i^ (149°)]; (**d**) C14–H14B···O6^i^ [C14–H14B (0.99 Å), C14 ···O6^i^ (3.251(7) Å), H14B···O6^i^ (2.37 Å), C14–H14B···O6^i^ (147°)]. Symmetry code: ^i^ = +x, +y, 1 + z.

An interesting structural feature of the compound [Cd(**L2**)NO_3_]NO_3_ is the hydrogen-bonding network (Figure 5) involving adjacent complex cation units and both nitrate anions. In particular, each coordinated NO_3_*^−^* anion forms a CH···O hydrogen bond with the macrocyclic ligand of a neighbouring [Cd(**L2**)NO_3_]^+^ cation complex, while each un-coordinated nitrate anion bridges two adjacent complex cations via NH···O and CH···O hydrogen bonds to form extended chains running along the *a*-axis.

In [Cu(**L2**)](ClO_4_)_2_, the stereoelectronic requirements of the metal centre are satisfied by the five donor atoms of the macrocyclic ligand, which imposes a N_3_S_2_ coordination sphere in a distorted trigonal bipyramidal geometry with the two S-donors in equatorial positions [Cu–N1 1.983(3), Cu–N2 2.109(3), Cu–N3 2.006(3), and Cu–S1 2.4697(11) and Cu–S2 2.3117(11) Å] (Figure 4). As expected, the Cu–N and Cu–S bond distances in [Cu(**L2**)]^2+^ are shorter than those observed in [Cd(**L2**)NO_3_]^+^ (due to the different ionic radii of the two metal ions) and similar to those observed in [Cu(**L1**)(NO_3_)_2_] [39].

**Figure 6:**
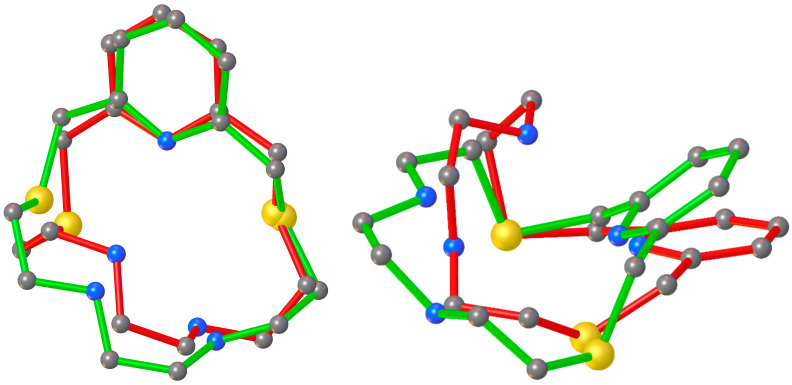
Superimposition of the structures of macrocyclic ligand **L2** in [Cu(**L2**)](ClO_4_)_2_·CH_3_CN (red) and [Cd(**L2**)(NO_3_)]NO_3_ (green).

Interestingly, the bond angles and the folded conformation assumed by **L2** in [Cd(**L2**)NO_3_]^+^ and [Cu(**L2**)]^2+^ are quite similar (Figure 6, Table S4 in the ESI), thereby indicating the absence of particular constraints upon coordination due to the different dimensions of the two metal ions, as well as the ability of **L2** to fully encapsulate both metal ions.

### 2.2. Synthesis of ***L3*** and Its Electrochemical Behaviour in the Presence of Cu^2+^, Zn^2+^, Cd^2+^, Hg^2+^, and Pb^2+^

The reaction of **L1** with (ferrocenylmethyl)trimethylammonium iodide in CH_3_CN in the presence of K_2_CO_3_ (anhydrous) afforded **L3** in 82% yield as an orange solid after standard work-up (see Section 3.5). Unfortunately, the insolubility of **L3** in aqueous solutions prevented a potentiometric study of its coordination properties towards the metal cations of interest, namely Cu^2+^, Zn^2+^, Cd^2+^, or Pb^2+^. However, it was possible to investigate by cyclic voltammetry the electrochemical response of **L3** to these metal cation species in the CH_2_Cl_2_/CH_3_CN 0.25:1 (*v*/*v*) solvent mixture (the most suitable for solubility reasons) at 25 °C. We were therefore able to unveil the effect on the response selectivity of this new chemosensor based on **L1** upon changing the transduction mechanism of the host-guest interaction (from optical to electrochemical) as compared to **L^a^**–**L^i^** (see above).

**Figure 7:**
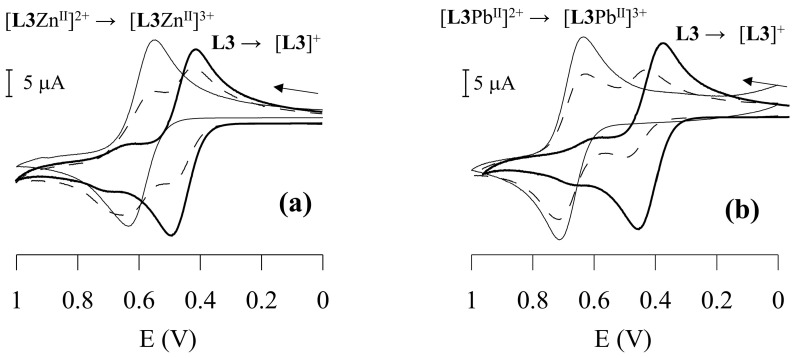
Cyclic voltammetry (scanned in the anodic direction) in CH_2_Cl_2_/CH_3_CN 0.25:1 (*v*/*v*) solvent mixture at 25 °C of: **L3** + Zn^2+^ (**a**), and **L3** + Pb^2+^ (**b**). The full bold line refers to free **L3**, the dashed line to **L3** + 0.5 equivs. of the metal ions, and the full thin line to **L3** + 1 equiv. of the metal ions. Scan rate 100 mV/s.

The cyclic voltammogram (scanned in the anodic direction with a scan rate of 100 mV/s) of the free ligand **L3** reveals a one-electron reversible (*i*_a_ ≈ *i*_c_) redox process at E_½_ = 450 mV vs. Ag/AgCl [calculated from the average of the oxidation (E_p_^ox^) and reduction (E_p_^red^) peak potentials], corresponding to the Fc^+^/Fc redox couple. While one-wave electrochemical behaviour is observed upon addition of HClO_4_ to the **L3** solution (due to the protonation of the macrocyclic moiety with an anodic shift of the Fc^+^/Fc redox couple of 190 mV), a two-wave behaviour is observed upon addition of increasing amounts of Cu^2+^, Zn^2+^, Cd^2+^, Pb^2+^ or Hg^2+^. The oxidation wave corresponding to the Fc^+^/Fc redox couple in free **L3** is gradually replaced by a new reversible wave at more positive potentials upon addition of increasing amounts of the metal ions, corresponding to the Fc^+^/Fc redox couple of the [**L**M^II^]^2+^ species. Its anodically shifted position with respect to the wave corresponding to un-complexed **L3** reflects a less favourable oxidation process for the Fc moiety in [**L**M^II^]^2+^ due to the presence of a positively charged metal cation centre bound to the receptor moiety, in close proximity. The currents for the new reversible redox couple [**L**M^II^]^3+^/[**L**M^II^]^2+^ (M = Cu^2+^, Zn^2+^, Cd^2+^, Pb^2+^ or Hg^2+^) increase linearly until one equiv. of the metal cation species is added. At this point, the reversible oxidation wave corresponding to un-complexed **L3** disappears (Figure 7).

Two-wave electrochemical behaviour in the cyclic voltammogram has been already observed for redox chemosensors featuring ferrocenyl signalling unit(s) upon addition of a metal ion, and can be accounted for by a high stability constant of the complex between the metal ion guest and the unoxidised redox responsive ionophore, and a large difference between the half-wave potentials for the two redox couples [**L3**]^+^/**L3** and [**L3**M^II^]^3+^/[**L3**M^II^]^2+^, with the magnitude of the anodic shift being related to the guest’s polarising power or its charge density [14,48,49,50,51,52,70]. In particular, by coupling in a square thermodynamic scheme the equilibria for the electrode oxidation of the neutral ionophore and its metal complexes with the equilibra for the metal guest complexations of the ionophore in its neutral and oxidised forms, the following equation can be derived [14,48]:

ΔE = *E*_½_^complex^ − *E*_½_^free ionophore^ = (RT/nF)ln (*K*_red_/*K*_ox_) (1)

where *K*_red_ and *K*_ox_ are the metal binding constants for the neutral and oxidised forms, respectively, of the redox chemosensor. The ratio *K*_red_/*K*_ox_ was defined by Beer [57] as reaction coupling efficiency (RCE). Its reciprocal is often referred to as the binding enhancement factor (BEF), and together with the anodic shift in the oxidation potential produced by presence of a metal cation (*E*_1/2_^complex^ − *E*_1/2_^free ionophore^), represents a quantitative measure of the perturbation of the redox centre induced by the guest complexation to the receptor unit (the perturbation is mainly electrostatic via through-space interaction); it allows evaluation of the efficiency of the signalling pathway that couples the guest binding event by the receptor unit, and the oxidation process of the ferrocenyl signalling unit, and gives a measure of the response selectivity of the redox chemosensor [60].

The largest redox shift ΔE_½_ (E_½_^complex^ *−* E_½_^free **L3**^) of 230 mV is observed in the presence of Pb^2+^ (Table 1) with a value of 7.9 × 10^3^ for RCE, which means that in CH_3_CN solution, **L3** binds Pb^2+^ ca. 7–8 × 10^3^ times more strongly than its oxidised form [**L3**]^+^. Significantly lower RCE and redox shift values are observed for all other metal ions (Table 1).

In light of these results, we investigated the coordination of **L3** to Pb^2+^ by DFT calculations. The geometry of the 1:1 complex [Pb(**L3**)]^2+^ was optimised at the DFT level (mPW1PW functional [71]; Def2SVP basis set [72]) along with the explicitly solvated species [Pb(**L3**)(CH_3_CN)*_n_*]^2+^ (*n* = 1–4). In all species, the Pb^2+^ ion is displaced from the ring cavity and coordinated by the S and N atoms of the macrocyclic unit, which adopts a folded conformation recalling that described above for [Pb(**L1**)(ClO_4_)_2_]·½CH_3_CN. The CH_3_CN units complete the coordination of the metal ion (Figure S2 for [Pb(**L3**)(CH_3_CN)_3_]^2+^), with the ferrocenylmethyl pendant pointing outwards without a direct interaction with the Pb^2+^ ion. A natural bonding analysis [73] shows that the Wiberg bond indexes [74] of the Pb–S bonds are larger than those of the Pb–N ones, with the aliphatic N-atom binding the metal ion more strongly than the N-atom from the pyridine ring (Table S5). Accordingly, a second order perturbation theory analysis of the Fock matrix in the NBO basis [73] shows that in [Pb(**L3**)(CH_3_CN)_4_]^2+^ the lone pairs of electrons (LPs) on the S-atom of the macrocyclic moiety contribute to the bond with a charge-transfer energy as large as about 67 kcal·mol^−1^, while the LPs on the pyridine and the macrocycle N-atoms with about 34 and 26 kcal·mol^−1^, respectively. The energy corresponding to the LP(CH_3_CN) → BD*(Pb^2+^) is calculated in the range 30–35 kcal·mol^−1^. The effect of the progressive increase in coordinated CH_3_CN molecules is to weaken the interaction of the macrocycle donor atoms with the Pb^2+^ ion, simultaneously decreasing the net natural charge Q_Pb_ on the metal ion (Q_Pb_ = 1.231, 1.213, 1.188, and 1.158 |e| for *n* = 1, 2, 3, and 4, respectively, in [Pb(**L3**)(CH_3_CN)*_n_*]^2+^; Table S5). A thermochemical analysis of the resulting complexes demonstrates that the reaction free energy ΔG_r_ values of the partial reactions is negative for *n* = 1–4:

[Pb(**L3**)]^2+^ + CH_3_CN = [Pb(**L3**)(CH_3_CN)]^2+^          ΔG_r_ = –16.3 kcal·mol^−1^

[Pb(**L3**)(CH_3_CN)]^2+^ + CH_3_CN = [Pb(**L3**)(CH_3_CN)_2_]^2+^   ΔG_r_ = –7.7 kcal·mol^−1^

[Pb(**L3**)(CH_3_CN)_2_]^2+^ + CH_3_CN = [Pb(**L3**)(CH_3_CN)_3_]^2+^   ΔG_r_ = –9.47 kcal·mol^−1^

[Pb(**L3**)(CH_3_CN)_3_]^2+^ + CH_3_CN = [Pb(**L3**)(CH_3_CN)_4_]^2+^   ΔG_r_ = –4.31 kcal·mol^−1^

Accordingly, the global ΔG_r_ for the reactions [Pb(**L3**)]^2+^ + n CH_3_CN = [Pb(**L3**)(CH_3_CN)*_n_*]^2+^. (*n* = 1–4) is calculated to be exoergonic by –16.23, –23.97, –33.44, and –37.74 kcal·mol^−1^ for *n* = 1, 2, 3, and 4, respectively. These results support that the Pb^2+^ ion can be successfully coordinated by the ligand **L3** in solution, even in the absence of an anion directly participating to coordination as in the structure of [Pb(**L1**)(ClO_4_)_2_]·½CH_3_CN, and that the solvent molecules CH_3_CN actively contribute to the solvation of the Pb^2+^ ion (with average Wiberg bond indexes of 0.162 for n = 1; 0.158 for n = 2; 0.147 for n = 3; 0.141 for n = 4; Table S5).

The electrochemical behaviour of **L3** in the presence of the metal cations considered indicates that the macrocyclic ligand **L1** bound to the ferrocenyl group can act as receptor unit in a redox-switchable chemosensor, with the highest shift for *E*_½_ observed in the presence of Pb^2+^. This result is not consistent with the electrochemical response predictable from the low charge density of Pb^2+^ as compared to the other metal cations considered. Previous studies on crown ethers and polyaza macrocycles linked to ferrocenyl group(s) via a methylene linker suggest a linear correlation between the observed Δ*E*_½_ values and the charge density of the complexed cations (in particular for the elements belonging to the first two groups of the periodic table). The electrostatic coupling (repulsion) between the complexed metal cation and the positive charge on the oxidised form of the ferrocenyl unit (either via through-bond or through-space) forms the basis of the mechanism responsible for the electrochemical recognition in these systems [14,48,51,52,75,76].

However, a comparison of the results obtained with **L3** with those reported for similar Fc-based redox chemosensors in similar solvent media could give valuable and more general indications. In fact, on considering 12-membered tetracoordinating macrocyclic ligands like **L1** linked to a ferrocenyl group through a methylene spacer, and containing donor atoms other than oxygen in the macrocyclic moieties, an interesting trend is observed. For example, the ligand Fc–CH_2_–([12]aneN_4_) shows a linear increase in *E*_½_ from 0.596V (Cd^2+^) to 0.659 (Co^2+^) vs. the charge density of the considered metal cations [Cd^2+^ < Zn^2+^ < Ni^2+^ < Co^2+^ < Cu^2+^; measurements were made in CH_2_Cl_2_/CH_3_CN 1:4 (*v*/*v*) solutions]. However, the data point for Ni^2+^ does not lie on the straight correlation line of the plot showing an *E*_½_ lower than expected (0.593 V) from the basis of the large charge density of the metal ion [52].

**Figure 8:**
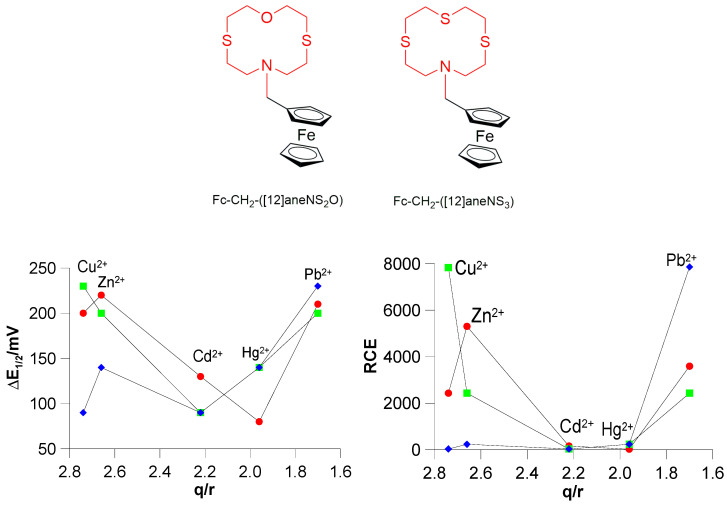
Scatter plot of Δ*E*_½_ = (*E*_½_^complex^ − *E*_½_^free **ligand**^) vs. q/r (bottom left) and RCE vs. q/r (bottom right) for **L3** (Table 1) (♦), Fc–CH_2_–([12]aneNS_3_) (■), and Fc–CH_2_–([12]aneNS_2_O) (●) [70] in the presence of Cu^2+^, Zn^2+^, Cd^2+^, Hg^2+^, and Pb^2+^ (q = +2; r = atomic radius of the metal cation). For Fc–CH_2_–([12]aneNS_3_) (■), and Fc–CH_2_–([12]aneNS_2_O) (●) measurements were made in CH_3_CN [70].

Even more interesting is the comparison with Fc-based redox chemosensors featuring sulphur among the donor atoms in the macrocyclic receptor unit. In Figure 8, the scatter plot of ΔE_½_ and RCE values vs. the ionic potential q/r (q = +2, r = radius of the metal cation in an octahedral environment [77]), which is equivalent to the charge density, is reported for **L3** and the structurally analogues Fc–CH_2_–([12]aneNS_2_O) and Fc–CH_2_–([12]aneNS_3_). These ligands, which feature-mixed N/S/O- and N/S-donor sets, respectively, in the macrocyclic moiety, result from the replacement of the pyridine moiety in **L3** with an oxygen or a sulphur atom [70], respectively, and are characterised by reduced conformational constraints. Both scatter plots clearly demonstrate the absence of a linear correlation between ΔE_½_/RCE and the ionic potential (q/r) of the metal cations with **L3** being unexpectedly highly selective for Pb^2+^. Note that Pb^2+^ displays the lowest charge density among the considered metal cations, and Fc–CH_2_–([12]aneNS_2_O) and Fc–CH_2_–([12]aneNS_3_) show the highest shift for *E*_½_ (and consequently the highest RCE value) in the presence of Zn^2+^ (Δ*E*_½_ = 220) and Cu^2+^ (Δ*E*_½_ = 230 mV), respectively, as would be expected on the basis of their higher charge density. However, for all three ligands a significant shift of *E*_½_ is observed in the presence of Pb^2+^ in the order **L3** (230 mV) > Fc–CH_2_–([12]aneNS_2_O) (210 mV) > Fc–CH_2_–([12]aneNS_3_) (200 mV). In general, as the charge density or ionic potential increases, a greater via through-space electrostatic repulsion between the oxidised form of the chemosensor [**L**]^+^ and the metal cation coordinated to the receptor unit is expected, resulting in a lower *K*_ox_ value. A high *K*_red_/low *K*_ox_ situation for high charge density metal ions would imply a high selectivity in the electrochemical response of redox chemosensors featuring ferrocene as the active unit, such as **L3**.

Considering the above, the fact that the three ligands featuring 12-membered mixed donor macrocyclic receptors, in particular **L3**, exhibit a significant electrochemical response for Pb^2+^ in terms of measured Δ*E*_½_ and RCE values (Figure 8) [despite *K*_red_ with this metal cation being lower than those with Cu^2+^, although slightly higher than those with Zn^2+^, see above], suggests that *K*_ox_ with Pb^2+^ is much lower than expected based only on the electrostatic repulsion with the ligands in the oxidised form. Clearly, under the commonly considered and accepted hypothesis of a sensing mechanism mainly based on an electrostatic via through-space interaction, a fine modulation of this interaction by other factors come into play and become dominant especially in the case of transition and post-transition metal cations (indeed, a linear dependence of Δ*E*_½_ and RCE on parameters such as the charge density or the ionic potential appears not to hold for these metal ions), thus dramatically affecting the selectivity of Fc-based redox chemosensors predictable on basis of only the metal cation charge density. Although it is difficult to identify all factors playing a role in the sensing mechanism of Fc-based redox chemosensors, as well as to quantify their effects, in the case of transition metal cations the coordination environment imposed by their electronic requirements, as well as the hard–soft nature of the donor atoms in the macrocyclic receptor unit of the chemosensor, its conformational properties, and finally the nature of the solvent can also be considered to play a very important and crucial role in determining a favourable balance between the thermodynamic selectivities of the host–guest interactions (*K*_red_ and *K*_ox_) for a net electrochemical response selectivity. Demonstrating the high number of factors beyond the charge density of the metal cation guest, and their complex interconnection in determining the unpredictable response selectivity of Fc-based redox chemosensors, the case of the analogous ionophore 1-ferrocenylmethyl 1-aza-4,7,10-trioxacyclododecane (Fc–CH_2_–([12]aneNO_3_), which has an NO_3_ donor set in the 12-membered macrocyclic receptor unit, is quite emblematic. It exhibits a selective electrochemical response in terms of the largest redox shift ΔE_½_ measured in CH_3_CN solution to the heavy and soft metal ions Hg^2+^ and Pb^2+^ [78].

## 3. Materials and Methods

### 3.1. Reagents and Apparatus

All chemicals used, including solvents and metal salts, were analytical reagent-grade; they were purchased from commercial sources where available (Sigma or Merck, Darmstadt, Germany) and used without any further purification. **L1** [39], **L2** [41], and (ferrocenylmethyl)trimethylammonium iodide [79] were prepared according to procedures reported in the literature. Microanalytical data were obtained using a Fisons EA CHNS-O (Fisons, Loughborough, UK) instrument operating at 1000 °C. ^1^H- and ^13^C-NMR spectra were recorded on a Varian VXR300 or a Varian VXR600 spectrometer (as specified below) (Varian, Inc., Palo Alto, CA, USA) and the chemical shifts were referred to the signals of the solvent. The FTIR spectra were recorded on a Thermo-Nicolet 5700 spectrometer (Thermo Electron Corporation, Madison, WI, USA) on KBr pellets, by using a KBr beam splitter out and KBr windows (4000–400 cm^−1^, resolution 4 cm^−1^). Cyclic voltammetry experiments were recorded on a computer-controlled EG&G (Princeton Applied Research, AMETEK Scientific Instruments, Oak Ridge, TN, USA) potentiostat-galvanostat Model 273 EG&G, using model 270 electrochemical analysis software. The mass spectra were recorded in the *m*/*z* 100–1000 range on a triple quadrupole QqQ Varian 310-MS mass spectrometer (Varian, Inc., Palo Alto, CA, USA) by using the atmospheric pressure ESI technique. Sample solutions (CH_3_CN) were infused into the ESI source with a programmable syringe pump (1.50 mL/h constant flow rate). The mMass 5.5.0 software package [80] was used for analysing the isotopic patterns of the peaks recorded in the mass spectra.

### 3.2. Procedure for Cyclic Voltammetry Experiments

Cyclic voltammetry experiments were conducted at 25 °C in anhydrous solvents [CH_2_Cl_2_/CH_3_CN 0.25:1 (*v*/*v*)] using a conventional three-electrode cell, consisting of a combined working and counter platinum electrode and a standard Ag/AgCl (in KCl 3.5 mol·dm^−3^; standard reduction potential 0.2223 V at 25 °C) reference electrode (scan rate of 100 mV/s). The solutions were 2.28 × 10^−3^ mol·dm^−3^ in the electroactive species **L3** with ^n^Bu_4_NBF_4_ (0.1 mol·dm^−3^) as supporting electrolyte. A stream of argon was passed through the solution prior to the scan. Different solutions were prepared for **L3** containing an increasing amount of the metal guest cation as hydrated perchlorate or nitrate salt (molar ratio ranging from 0 to 1:1), and the cyclic voltammogram was recorded for each solution. The titration of the ligands with HClO_4_ was performed by cautiously adding μL amounts of the concentrated acid (1 mol·dm^−3^) to the solution of the electroactive species.

### 3.3. X-Ray Crystallography

Single-crystal X-ray diffraction data for [Pb(**L1**)(ClO_4_)_2_]·½CH_3_CN and [Cu(**L2**)](ClO_4_)_2_·CH_3_CN were collected at 293 K on a Bruker APEX II CCD diffractometer using ω scans. Data reduction and processing were carried out using SAINT [81] and SADABS [82]. The structures were solved by dual methods using SHELXT [83], and the models were refined through iterative cycles of least-squares refinement on *F*^2^ with SHELXL [84].

For compound [Pb(**L1**)(ClO_4_)_2_]·½CH_3_CN, two perchlorates [those belonging to unit **A** (see above)] were disordered and modelled over two sites using geometric restraints for Cl–O distances and constraining the atomic displacement parameters of O atoms to be the same.

One disordered acetonitrile molecule per asymmetric unit could not be modelled, and its electron density was accounted by using the SQUEEZE routine implemented in Platon [85].

For compound [Cu(**L2**)](ClO_4_)_2_·CH_3_CN, a perchlorate was disordered and modelled over two positions with fractional occupancies 62:38, respectively.

Single-crystal X-ray diffraction data for [Cd(**L2**)(NO_3_)]NO_3_ were collected at 150 K on a Bruker SMART APEX CCD diffractometer. The structure was refined as a two-component inversion twin, solved by direct methods using SHELXS [86], and the model developed with SHELXL [84]. Olex2 [87] was used as the graphical interface for structure solution and refinement and for the preparation of figures.

All non-H atoms were refined anisotropically. H atoms were introduced at calculated positions and thereafter incorporated into a riding model with *U*_iso_(H) = 1.2*U*_eq_(C).

### 3.4. DFT Calculations

Theoretical calculations were carried out on **L3**, CH_3_CN, and the model compounds [Pb(**L3**)]^2+^ and [Pb(**L3**)(CH_3_CN)*_n_*]^2+^ (*n* = 1–4) at the density functional theory (DFT) level with the commercial suite of programs Gaussian 16 [88], adopting the mPW1PW hybrid functional [71], and the Def2SVP [72] basis set, including pseudopotential parameters for the heavier Pb atom (Tables S5–S11). Harmonic frequency calculations were carried out at the optimised geometries to check the nature of the energy minima by verifying the absence of significative negative frequencies. Free energy variations ΔG_r_ associated with the solvation reactions were calculated as differences of the sum of electronic and thermal free energies calculated for products and reactants. All thermochemical calculations assumed T = 298.15 K as a part of harmonic frequency calculations. Natural charge distributions [73] and Wiberg [74] bond indexes (Table S5) were calculated at the same level of theory. The program GaussView 6.0.16 [89] was used to investigate the optimised structures and natural charge distribution.

### 3.5. Synthesis of 5-Ferrocenylmethyl-2,8-dithia-5-aza-2,6-pyridinophane (***L3***)

A solution of (ferrocenylmethyl)trimethylammonium iodide (1.21 g, 3.15 mmol) in anhydrous CH_3_CN (50 mL) was added dropwise to a refluxing solution of **L1** (0.5 g, 2.08 mmol) and K_2_CO_3_ (2.32 g, 16.8 mmol) in anhydrous CH_3_CN (40 mL). The resulting orange solution was stirred overnight at 80 °C under an atmosphere of N_2_. K_2_CO_3_ was then filtered off and the solvent was removed under reduced pressure. The residue was taken up in CH_2_Cl_2_ and washed with water. The organic extracts were dried over Na_2_SO_4_, filtered, and the solvent removed under reduced pressure to give the desired compound as an orange solid (yield 0.75 g, 1.7 mmol, 82%). Mp.: 135 °C. Anal. found (calc. for C_22_H_26_FeN_2_S_2_): C, 59.88 (60.27); H, 6.15 (5.98); N, 6.60 (6.39); S, 14.60 (14.63) %. ^1^H-NMR (600 MHz, CDCl_3_, Figure S3): *δ*_H_ 2.55 (m, 8H), 3.30 (s, 2H), 3.80 (s, 4H), 4.07 (s, 5H), 4.30 (s, 4H), 7.25 (d, 2H, J = 12 Hz), 7.68 ppm (t, 1H, J = 6.0 Hz). ^13^C-NMR (150 MHz, CDCl_3_, Figure S4): *δ*_C_ 25.5, 36.6, 51.1, 52.7, 67.3, 68.5, 69.6, 70.7, 72.1, 121.6, 138.2, 157.7 ppm. ESI(+) MS (CH_3_CN solution, Figure S5) *m*/*z*: 439.1 ([C_22_H_26_FeN_2_S_2_]^+^).

### 3.6. Synthesis of [Pb(***L1***)(ClO_4_)_2_]·½CH_3_CN

To a solution of **L1** (0.020 g, 0.083 mmol) in CH_3_CN (10 mL) was added Pb(ClO_4_)_2_·3H_2_O (0.038 g, 0.083 mmol) dissolved in CH_3_CN (2 mL). The mixture was stirred at room temperature under N_2_ for several hours. Colourless micro crystals (yield 0.05 g, 45%) were obtained by diffusion of Et_2_O vapour into the CH_3_CN solution. Mp: 250 °C with decomposition. Elem. Anal. found (calc. for C_12_H_17.5_Cl_2_N_2.5_O_8_PbS_2_): C, 21.70 (21.61); H, 2.48 (2.64); N, 5.22 (5.25); S, 9.50 (9.61) %. FT-IR (KBr): ν = 3508 (w,br), 3251 (m), 2978 (w), 2932 (w), 1595 (m), 1449 (m), 1240 (w), 1090 (s,br), 999 (w), 785 (w), 623 (m) cm^−1^. ^1^H-NMR (600 MHz, CD_3_CN): *δ*_H_ 2.55 (m, 2H), 3.73 (m, 2H), 3.09 (m, 2H), 3.50 (m, 2H), 4.08 (d, 2H, J = 16.9 Hz), 4.61 (d, 2H, J = 16.9 Hz), 7.34 (d, 2H, J = 6.6 Hz), 7.83 ppm (t, 1H, J = 8.0 Hz). ^13^C-NMR (150 MHz, CD_3_CN): *δ*_C_ 31.7, 38.0, 52.1, 126.9, 141.5, 161.4 ppm (aromatic carbons). ESI(+) MS (CH_3_CN solution) *m*/*z*: 448.3 ([C_11_H_16_N_2_PbS_2_]^+^).

### 3.7. Synthesis of [Cu(***L2***)](ClO_4_)_2_·CH_3_CN

To a solution of **L2** (0.020 g, 0.070 mmol) in CH_3_CN (10 mL) was added Cu(ClO_4_)_2_·2H_2_O (0.021 g, 0.070 mmol) dissolved in EtOH (2 mL). The mixture was stirred at room temperature under N_2_ for 2 h. Blue crystals (yield 0.025 g, 61%) were obtained by diffusion of Et_2_O vapour into the reaction mixture. Mp: 163° C with decomposition. Elem. Anal. found (calc. for C_15_H_24_Cl_2_CuN_4_O_8_S_2_): C, 30.55 (30.69); H, 3.88 (4.12) N, 9.32 (9.54); S, 10.54 (10.92) %. FT-IR (KBr): ν = 3436 (m,br), 1644 (m), 1462 (w), 1384 (w), 1087 (s), 810 (w), 627(m) cm^−1^. ESI(+) MS (CH_3_CN solution) *m*/*z*: 445.8 ([C_13_H_21_ClCuN_3_O_4_S_2_]^+^).

### 3.8. Synthesis of [Cd(***L2***)NO_3_]NO_3_

To a solution of **L2** (0.020 g, 0.070 mmol) in CH_3_CN (10 mL) was added Cd(NO_3_)_2_·4H_2_O (0.022 g, 0.070 mmol) dissolved in CH_3_CN (2 mL). The mixture was stirred at 50 °C under nitrogen for 24 h. Colourless crystals (yield 0.023 g, 62%) were obtained by diffusion of Et_2_O vapour into CH_3_CN solution. Mp: 230 °C with decomposition. Elem. Anal. found (calc. for C_13_H_21_CdN_5_O_6_S_2_): C, 30.39 (30.03); H, 4.25 (4.07) N, 13.40 (13.47); S, 12.45 (12.34) %. FT-IR (KBr): ν = 3448 (w,br), 2917 (w), 1624 (m), 1384 (m), 1120 (s), 620 (w) cm^−1^. ESI(+) MS (CH_3_CN solution) *m*/*z*: 459.2 ([C_13_H_21_CdN_4_O_3_S_2_]^+^).

## 4. Conclusions

In this paper, we report on the use of **L1** as a receptor unit in the construction of a conjugated redox chemosensor for heavy metal ions, **L3**, featuring a ferrocenyl signalling unit. Cyclic voltammetry experiments proved the compound to be selective for lead(II), showing the highest anodic shift for this metal ion, among the metal ions considered (Cu^2+^, Zn^2+^, Cd^2+^, Pb^2+^, and Hg^2+^), in reversible two-wave behaviour of the Fc^+^/Fc redox couple, in CH_2_Cl_2_/CH_3_CN 0.25:1 (*v*/*v*) solvent mixture at 25 °C. These results confirm the complex interplay of the many factors, beyond the charge density of the metal ion guests, in determining the signalling selectivity for transition metal cations of Fc-based redox chemosensors. To date, the major role of the electrostatic via through-space interactions on the selectivity of Fc-based redox chemosensors is not fully understood and quantifiable/rationalizable.

Besides this, we have also shown the great versatility of **L1** as a receptor unit (despite its low binding selectivity) in the design of conjugated chemosensors involving either an optic or electrochemical transduction mechanism, with a “synergism cooperation” between the receptor and signalling units determining at least a signalling selectivity towards different metal ions. The crystal structures of the compounds [Pb(**L1**)(ClO_4_)_2_]·½CH_3_CN, [Cu(**L2**)](ClO_4_)_2_·CH_3_CN and [Cd(**L2**)(NO_3_)]NO_3_ have allowed us to gain a deeper knowledge of the coordination properties of **L1** in comparison with those of **L2**.

## Figures and Tables

**Table 1:** Electrochemical data of **L3** upon addition of H^+^, Cu^2+^, Zn^2+^, Cd^2+^, Hg^2+^, and Pb^2+^ as determined by cyclic voltammetry ^a^.

Cation	*E*_½_^complex^/mV	Δ*E*_½_/mV ^b^	RCE (*K*_red_/*K*_ox_) ^c^
H^+^	640	190	^---^
Cu^2+^	540	90	33
Zn^2+^	590	140	235
Cd^2+^	540	90	33
Hg^2+^	590	140	235
Pb^2+^	680	230	7.9 × 10^3^

^a^ Data obtained at 25 °C in a CH_2_Cl_2_/CH_3_CN 0.25:1 (*v*/*v*) solvent mixture containing 0.1 M ^n^Bu_4_NBF_4_ as supporting electrolyte, vs. Ag/AgCl standard electrode, and a platinum working electrode; [**L3**] = 2.28 × 10^−3^ M; ^b^ shift in the oxidation potential produced by presence of the metal ion guest (0.5 equivalents); *E*_½_^free **L3**^ = 450 mV; ^c^ reaction coupling efficiency (RCE), which can be calculated only in the two-wave case. E_½_^complex^ = (E_p_^ox^)^complex^ − (E_p_^red^)^complex^; ΔE_½_ = E_½_^complex^ − E_½_^free **L3**^.

## Data Availability

The raw data supporting the conclusions of this article will be made available by the authors on request. Crystallographic data have been deposited with the Cambridge Crystallographic Data Centre (CCDC) under deposition no. 2391106 ([Pb(**L1**)(ClO_4_)_2_]·½CH_3_CN), 2391107 ([Cd(**L2**)(NO_3_)]NO_3_), 2391105 ([Cu(**L2**)](ClO_4_)_2_·CH_3_CN). These data can be obtained free charge at: https://www.ccdc.cam.ac.uk/structures.

## Supplementary Materials

The following supporting information can be downloaded at: https://www.mdpi.com/article/10.3390/molecules30010130/s1, Figure S1: Distribution of the Pb–O(ClO_4_^−^) distances retrieved from the Cambridge Crystallographic Database (CSD); Figure S2: Structure of the model complex [Pb(**L3**)(CH_3_CN)_3_]^2+^ optimised at DFT level; Figure S3: ^1^H-NMR spectrum of **L3** in CDCl_3_; Figure S4: ^13^C-NMR spectrum of **L3** in CDCl_3_; Figure S5: ESI(+) MS of **L3** in CH_3_CN; Table S1: Crystallographic data and refinement parameters for [Pb(**L1**)(ClO_4_)_2_]·½CH_3_CN, [Cu(**L2**)](ClO_4_)_2_·CH_3_CN, and [Cd(**L2**)(NO_3_)]NO_3_; Table S2: Selected bond and distances (Å) and angles (°) for [Pb(**L1**)(ClO_4_)_2_]·½CH_3_CN; Table S3: Pb–O distances (Å) and selected O–Pb–O angles (^o^) in [Pb(**L1**)(ClO_4_)_2_]·½CH_3_CN; Table S4: Selected bond lengths (Å) and angles (°) for [Cu(**L2**)](ClO_4_)_2_·CH_3_CN and [Cd(**L2**)(NO_3_)]NO_3_; Table S5: Selected bond lengths (Å) and corresponding Wiberg bond indexes (in parentheses) calculated for the complexes [Pb(**L3**)(CH_3_CN)*_n_*]^2+^ (*n* = 1–4) at the DFT-optimised geometry; Tables S6–S11: Optimised geometry calculated at DFT level for CH_3_CN, [Pb(**L3**)]^2+^, [Pb(**L3**)(CH_3_CN)*_n_*]^2+^ (*n* = 1–4), respectively.
