# Supplementary material for: Coordination Chemistry of Mixed-Donor Pyridine-Containing Macrocyclic Ligands: From Optical to Redox Chemosensors for Heavy Metal Ions
Source: Molecules. 2024 Dec 31;30(1):130. doi: 10.3390/molecules30010130 (PMC11722060; doi:10.3390/molecules30010130)
Supplement: Supplementary file 1 [file molecules-30-00130-s001.zip › molecules-3373726-supplementary.pdf]

Coordination Chemistry of Mixed-Donor Pyridine-Containing Macrocyclic Ligands: from Optical to Redox Chemosensors for Heavy Metal Ions

Alessandra Garau *, Alexander J. Blake, M. Carla Aragoni, Massimiliano Arca, Claudia Caltagirone, Francesco Demartin, Vito Lippolis *, Giacomo Picci, and Enrico Podda

SUPPORTING INFORMATION

Table S1. Crystallographic data and refinement parameters for [Pb(**L1**)(ClO_4)$_2$]·½MeCN, [Cu(**L2**)](ClO_4)$_2$·MeCN and [Cd(**L2**)(NO_3)]NO_3.

Compound	[Pb(**L1**)(ClO_4)$_2$]·½MeCN	[Cu(**L2**)](ClO_4)$_2$·MeCN	[Cd(**L2**)(NO_3)]NO_3
Empirical formula	$C_{12}H_{17.5}Cl_2N_{2.5}O_8PbS_2$	$C_{15}H_{23}Cl_2CuN_4O_8S_2$	$C_{13}H_{21}CdN_5O_6S_2$
Formula weight	666.99	585.93	519.87
Temperature/K	293(2)	293(2)	150(2)
Crystal system	monoclinic	triclinic	orthorhombic
Space group	$C2/c$	$P-1$	$Pna2_1$
a/Å	33.830(2)	9.9443(8)	15.134(2)
b/Å	9.217(4)	11.0898(9)	15.144(2)
c/Å	31.5780(10)	12.7135(11)	8.3953(11)
α/°	90	66.380(10)	90
β/°	122.150(10)	88.210(10)	90
γ/°	90	67.230(10)	90
Volume/Å^3	8336(4)	1171.7(2)	1924.1(4)
Z	16	2	4
ρ_{calc}g/cm^3	2.126	1.661	1.795
μ/mm^{-1}	8.594	1.387	1.393
Crystal size/mm^3	0.22 × 0.13 × 0.07	0.20 × 0.13 × 0.06	0.40 × 0.24 × 0.10
Radiation	Mo$K\alpha$ (λ = 0.71073)	Mo$K\alpha$ (λ = 0.71073)	Mo$K\alpha$ (λ = 0.71073)
2θ range for data collection/°	2.844 to 50.698	3.534 to 52.084	5.38 to 55.114
Reflections collected	28697	8109	8490
Independent reflections	7630 [R_{int} = 0.0797]	4418 [R_{int} = 0.0238]	4345 [R_{int} = 0.035]
Data/restraints/parameters	7630/10/453	4418/10/336	4345/1/245
GooF on F^2	0.920	1.038	1.040
Final R indexes [I ≥ 2σ (I)]	R_1 = 0.0392<br>wR_2 = 0.0866	R_1 = 0.0442<br>wR_2 = 0.1190	R_1 = 0.0286<br>wR_2 = 0.0677
Final R indexes [all data]	R_1 = 0.0777<br>wR_2 = 0.0966	R_1 = 0.0604<br>wR_2 = 0.1270	R_1 = 0.0311<br>wR_2 = 0.0689
Largest diff. peak/hole / e Å^{-3}	1.29/−0.72	0.55/−0.39	1.01/−0.32
Flack parameter			−0.01(4)

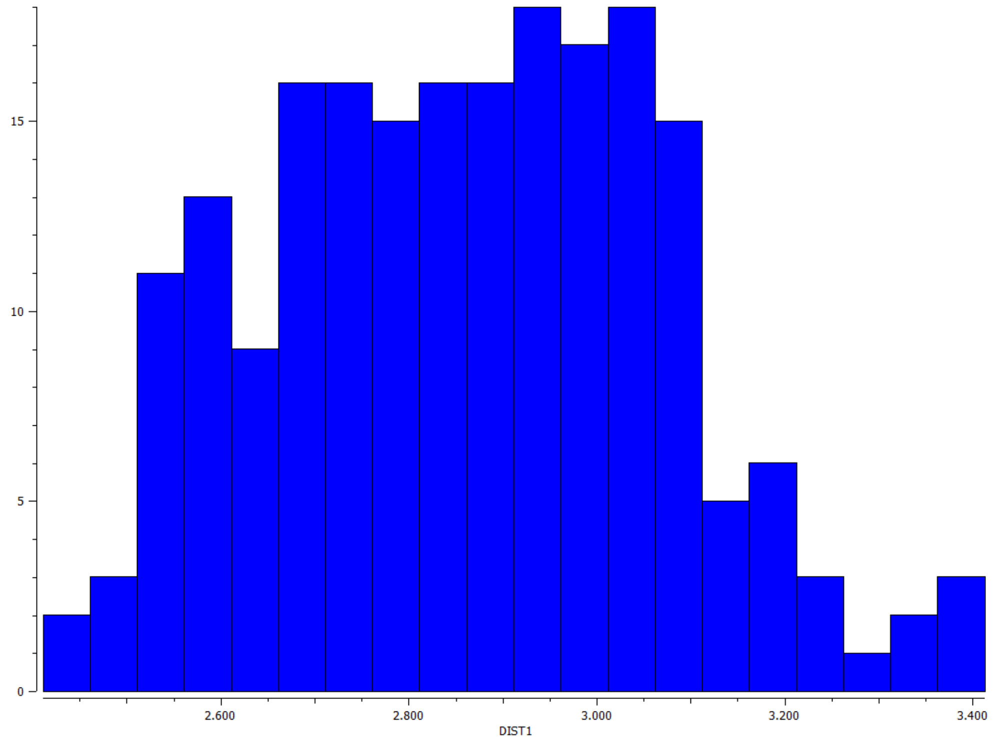

Figure S1. Distribution of the Pb–O(ClO_4^-) distances retrieved from the Cambridge Crystallographic Database (CSD) version 5.45 accessed on 20th of august 2024.

Table S2. Selected bond and distances (Å) and angles (°) for [Pb(**L1**)(ClO_4)$_2$]·½MeCN.

Pb1–S1	2.846(2)	Pb2–S4	2.866(2)
Pb1–S2	2.860(2)	Pb2–S3	2.878(2)
Pb1–N1	2.529(6)	Pb2–N3	2.542(7)
Pb1–N2	2.558(7)	Pb2–N4	2.566(7)
S1–Pb1–S2	130.12(8)	N3–Pb2–N4	82.5(2)
N1–Pb1–S1	71.9(2)	N4–Pb2–S4	72.3(2)
N1–Pb1–S2	70.8(2)	N4–Pb2–S3	70.5(2)
N1–Pb1–N2	83.5(2)	N3–Pb2–S3	71.5(2)
N2–Pb1–S1	71.7(2)	N3–Pb2–S4	71.6(2)
N2–Pb1–S2	72.1(2)	S3–Pb2–S4	129.97(9)

Table S3. Pb–O distances (Å) and selected O–Pb–O angles (º) in [Pb(**L1**)(ClO_4)$_2$]·½MeCN.

Pb1–O9A	2.954(15)	Pb2–O3	2.822(7)
Pb1–O11A	2.78(3)	Pb2–O4	3.059(11)
Pb1–O11B	2.81(2)	Pb2–O6	3.339(11)
Pb1–O13A^{i}	2.83(3)	Pb2–O7	2.999(7)
Pb1–O13B^{i}	3.32(3)	Pb2–O5^{ii}	3.294(11)
Pb1–O14A	2.89(2)		
Pb1–O14B	2.95(2)		
Pb1–O16A	3.294(18)		
Pb1–O16B	4.001(17)		
O9A–Pb1–O14A	134.6(6)	O3–Pb2–O6	105.0(2)
O9A–Pb1–O16A	98.5(4)	O3–Pb2–O7	136.0(2)
O11A–Pb1–O14A	114.5(8)	O4–Pb2–O6	77.3(2)
O11A–Pb1–O16A	75.4(6)	O4–Pb2–O7	118.9(3)

^{i} = 1−*x*, 1−*y*, 1−*z*; ^{ii} = 2−*x*, +*y*, 3/2−*z*.

Table S4. Selected bond lengths (Å) and angles (°) for [Cu(**L2**)]$(ClO_4)_2$·MeCN and [Cd(**L2**)(NO_3)]NO_3.

[Cu(**L2**)]$(ClO_4)_2$·MeCN		[Cd(**L2**)(NO_3)]NO_3	
Cu1–S1	2.4697(11)	Cd1–S1	2.7264(13)
Cu1–S2	2.3117(11)	Cd1–S2	2.7695(13)
Cu1–N1	1.985(3)	Cd1–N1	2.416(4)
Cu1–N2	2.109(3)	Cd1–N2	2.397(4)
Cu1–N3	2.006(3)	Cd1–N3	2.338(4)
		Cd1–O4	2.380(4)
		Cd1–O5	2.516(4)
S2–Cu1–S1	134.06(4)	S1–Cd1–S2	137.63(4)
N1–Cu1–S1	83.97(8)	N1–Cd1–S1	71.11(9)
N1–Cu1–S2	81.73(9)	N1–Cd1–S2	73.42(9)
N1–Cu1–N2	96.65(11)	N2–Cd1–S1	75.88(12)
N1–Cu1–N3	165.06(12)	N2–Cd1–S2	146.25(12)
N2–Cu1–S1	85.02(9)	N3–Cd1–S1	133.97(12)
N2–Cu1–S2	139.86(9)	N3–Cd1–S2	75.96(12)
N3–Cu1–S1	110.95(9)	N2–Cd1–N1	129.00(15)
N3–Cu1–S2	87.89(10)	N3–Cd1–N1	102.43(15)
N3–Cu1–N2	84.37(13)	N3–Cd1–N2	74.52(17)
		O4–Cd1–S1	88.15(11)
		O4–Cd1–S2	80.63(12)
		O4–Cd1–O5	51.84(13)
		O4–Cd1–N1	106.89(14)
		O4–Cd1–N2	109.77(17)
		O5–Cd1–S1	121.49(11)
		O5–Cd1–S2	82.18(11)
		N1–Cd1–O5	150.54(14)
		N2–Cd1–O5	80.32(16)
		N3–Cd1–O4	135.04(16)
		N3–Cd1–O5	87.00(15)

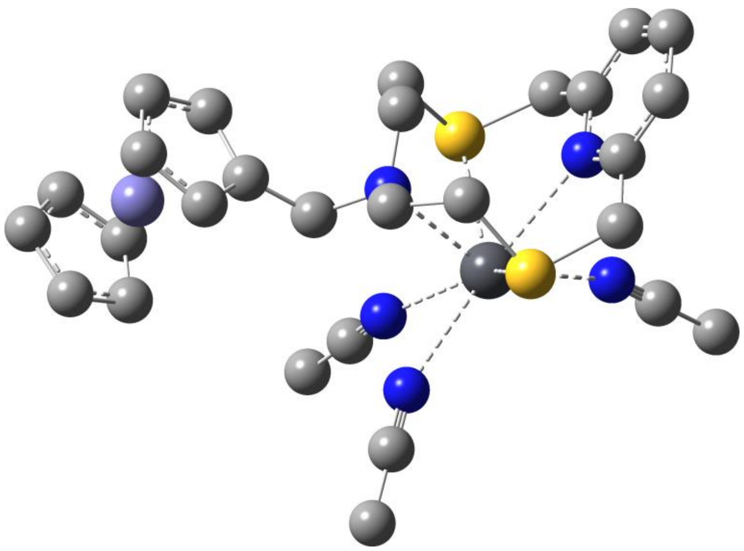

Figure S2. Structure of the model complex $[Pb(\mathbf{L3})(CH_3CN)_3]^{2+}$ optimized at DFT level. Hydrogen atoms were omitted for clarity.

Table S5. Selected bond lengths (Å) and corresponding Wiberg bond indexes (in parentheses) calculated for the complexes $[Pb(\mathbf{L3})(CH_3CN)_n]^{2+}$ (n = 1–4) at the DFT-optimized geometry.

	n = 1	*n* = 2	*n* = 3	*n* = 4
Pb–N_{Py}[a]	2.646	2.783	2.772	2.765
	(0.196)	(0.155)	(0.156)	(0.159)
Pb–N_{aza}[b]	2.532	2.557	2.640	2.734
	(0.265)	(0.256)	(0.197)	(0.160)
Pb–S	2.883 / 2.916	2.906 / 2.912	2.891 / 2.950	2.930 / 2.940
	(0.235 / 0.283)	(0.279 / 0.274)	(0.288 / 0.249)	(0.249 / 0.251)
Pb···N_{MeCN}[c]	2.761	2.766 / 2.804	2.846 / 2.916 / 2.880	2.899 / 2.916 / 2.945 / 2.974
	(0.162)	(0.160 / 0.156)	(0.154 / 0.147 / 0.141)	(0.145 / 0.143 / 0.139 / 0.136)

[a] Pyridine N-atom. b Macrocyclic N-atom. [c] N-atom of the acetonitrile units.

Table S6. Optimized geometry calculated for CH_3CN at DFT level (mPW1PW/Def2SVP) in the gas phase in orthogonal Cartesian coordinate format.

Center Number	Atomic Number	Coordinates (Angstroms) X	Y	Z
1	6	4.148422	-0.519647	0.000013
2	1	4.528568	-1.549935	-0.000031
3	1	4.528623	-0.004422	0.892219
4	1	4.528641	-0.004463	-0.892209
5	6	2.694931	-0.519573	0.000012
6	7	1.538950	-0.519526	-0.000004

Table S7. Optimized geometry calculated for $[Pb(\mathbf{L3})]^{2+}$ at DFT level (mPW1PW/Def2SVP) in the gas phase in orthogonal Cartesian coordinate format.

Center Number	Atomic Number	Coordinates (Angstroms) X	Y	Z
1	82	1.602517	-0.296787	-1.385514
2	16	2.311338	-2.606556	0.106948
3	7	3.478718	0.333627	0.198271
4	6	4.233163	-0.630783	0.765293
5	16	1.227871	2.433203	-0.672168
6	7	-0.011485	-0.304120	0.534758
7	6	5.255993	-0.322675	1.660650
8	1	5.846377	-1.122873	2.108280
9	6	5.511898	1.007713	1.972187
10	1	6.307576	1.272284	2.670725
11	6	4.727283	1.992547	1.386453
12	1	4.895591	3.045895	1.613376
13	6	3.716276	1.625354	0.497456
14	6	2.942027	2.732823	-0.171735
15	1	3.438557	3.015084	-1.116402
16	1	2.957499	3.635934	0.453342
17	6	0.295923	2.191491	0.876455
18	1	0.669212	2.914424	1.615827
19	1	-0.718590	2.519350	0.615633
20	6	0.300016	0.795282	1.474720
21	1	1.276458	0.587526	1.930773
22	1	-0.446896	0.791772	2.287712
23	6	0.024432	-1.601734	1.253640
24	1	-0.482980	-2.347525	0.624552
25	1	-0.577990	-1.524899	2.173030
26	6	1.407008	-2.113222	1.618587
27	1	1.303046	-3.008806	2.246660
28	1	2.006536	-1.383687	2.179603
29	6	4.018617	-2.079341	0.408685
30	1	4.448950	-2.727320	1.183514
31	1	4.547006	-2.332352	-0.526408
32	6	-2.526078	-0.095014	0.803013
33	6	-3.125033	1.075552	1.389787
34	6	-3.247799	-1.236767	1.299942
35	1	-2.857316	2.113499	1.197396
36	6	-4.187466	0.650674	2.232809
37	1	-3.077895	-2.277627	1.029146
38	6	-4.262014	-0.768684	2.177828
39	1	-4.853291	1.302452	2.793897
40	1	-4.994955	-1.387910	2.690015
41	26	-4.480253	0.030117	0.291974
42	6	-4.851832	-0.556541	-1.640355
43	1	-4.215792	-1.179694	-2.265905
44	6	-5.883943	-1.023085	-0.779624
45	1	-6.157450	-2.062469	-0.611403
46	6	-6.484604	0.107928	-0.160697
47	1	-7.293355	0.083397	0.566407
48	6	-4.815634	0.866246	-1.552574
49	1	-4.147081	1.528890	-2.098590
50	6	-5.825769	1.274783	-0.637674
51	1	-6.047939	2.297975	-0.342455
52	6	-1.363982	-0.132965	-0.128619
53	1	-1.468847	-0.975591	-0.829652
54	1	-1.325130	0.785350	-0.733264

Table S8. Optimized geometry calculated for $[Pb(\mathbf{L3})(CH_3CN)]^{2+}$ at DFT level (mPW1PW/Def2SVP) in the gas phase in orthogonal Cartesian coordinate format.

Center Number	Atomic Number	Coordinates (Angstroms) X	Y	Z
1	82	-1.662269	1.131957	-0.425879
2	16	-2.337681	1.067008	2.332908
3	7	-3.528809	-0.518491	-0.146431
4	6	-4.278364	-0.503070	0.973953
5	16	-1.283657	-0.908962	-2.432850
6	7	-0.029393	-0.505321	0.557410
7	6	-5.309144	-1.419802	1.179025
8	1	-5.895043	-1.385690	2.098367
9	6	-5.577464	-2.370886	0.202087
10	1	-6.378278	-3.099288	0.339813
11	6	-4.797124	-2.384734	-0.946231
12	1	-4.974721	-3.123356	-1.728894
13	6	-3.777571	-1.441674	-1.095782
14	6	-3.009825	-1.461585	-2.398009
15	1	-3.502084	-0.800873	-3.132388
16	1	-3.044118	-2.469063	-2.834327
17	6	-0.370663	-2.106344	-1.409462
18	1	-0.764913	-3.109765	-1.624553
19	1	0.642479	-2.080674	-1.830785
20	6	-0.366568	-1.872389	0.093616
21	1	-1.348469	-2.133178	0.508454
22	1	0.367970	-2.577510	0.520302
23	6	-0.067730	-0.463958	2.041491
24	1	0.457317	0.447085	2.363729
25	1	0.518113	-1.306506	2.442997
26	6	-1.450604	-0.495028	2.670693
27	1	-1.345692	-0.584231	3.760774
28	1	-2.062779	-1.339589	2.326793
29	6	-4.049793	0.547597	2.033812
30	1	-4.479018	0.216499	2.988542
31	1	-4.571491	1.484543	1.774627
32	6	2.469652	-0.912828	0.527329
33	6	3.033734	-2.034052	-0.176078
34	6	3.187440	-0.783865	1.768485
35	1	2.760599	-2.387769	-1.169090
36	6	4.068584	-2.582821	0.628227
37	1	3.040214	-0.015209	2.525469
38	6	4.162059	-1.815938	1.821530
39	1	4.705131	-3.422675	0.359122
40	1	4.882729	-1.969132	2.621636
41	26	4.446407	-0.615288	0.179328
42	6	4.911771	1.330945	-0.296584
43	1	4.317786	2.218549	-0.087636
44	6	5.909044	0.781979	0.555014
45	1	6.189980	1.155329	1.537413
46	6	6.465956	-0.352971	-0.096214
47	1	7.242270	-1.001244	0.304259
48	6	4.850003	0.531077	-1.476191
49	1	4.198167	0.692639	-2.332545
50	6	5.812927	-0.508596	-1.350356
51	1	6.008461	-1.293863	-2.077337
52	6	1.336753	-0.054454	0.076157
53	1	1.470007	0.974201	0.443407
54	1	1.305138	-0.002235	-1.022307
55	7	-0.374674	2.572357	-0.489173
56	6	0.404082	3.311796	-0.536856
57	6	1.580796	4.274959	-0.596849
58	1	1.643174	4.853090	0.310694
59	1	1.506880	4.937499	-1.443823
60	1	2.453547	3.650768	-0.693580

Table S9. Optimized geometry calculated for [Pb(**L3**)(CH_3CN)$_2$]$^{2+}$ at DFT level (mPW1PW/Def2SVP) in the gas phase in orthogonal Cartesian coordinate format.

Center Number	Atomic Number	Coordinates (Angstroms) X	Y	Z
1	82	-1.627218	1.148105	-0.302706
2	16	-2.556876	0.583887	2.392242
3	7	-3.627330	-0.786668	-0.246773
4	6	-4.363917	-0.970191	0.860765
5	16	-1.336828	-0.772241	-2.472171
6	7	-0.104702	-0.720632	0.549773
7	6	-5.296965	-2.003623	0.966179
8	1	-5.873441	-2.129226	1.883252
9	6	-5.478503	-2.863297	-0.110442
10	1	-6.198834	-3.680955	-0.054684
11	6	-4.717518	-2.665685	-1.255742
12	1	-4.831254	-3.321186	-2.119777
13	6	-3.801601	-1.611710	-1.290287
14	6	-3.042266	-1.375763	-2.571145
15	1	-3.552978	-0.602490	-3.169596
16	1	-3.044350	-2.284796	-3.187505
17	6	-0.440442	-2.075893	-1.570403
18	1	-0.821502	-3.050131	-1.908443
19	1	0.586407	-2.001590	-1.950364
20	6	-0.488555	-2.014318	-0.052897
21	1	-1.498871	-2.265003	0.290210
22	1	0.188391	-2.800064	0.326748
23	6	-0.193854	-0.810926	2.026418
24	1	0.284802	0.088733	2.437762
25	1	0.408432	-1.667916	2.370782
26	6	-1.587932	-0.949759	2.612640
27	1	-1.494739	-1.133621	3.691792
28	1	-2.151000	-1.791148	2.187262
29	6	-4.225469	-0.013861	2.017507
30	1	-4.653101	-0.453283	2.928189
31	1	-4.799082	0.907514	1.822762
32	6	2.377674	-1.249839	0.603307
33	6	2.900856	-2.373907	-0.124852
34	6	3.094542	-1.183136	1.847028
35	1	2.612515	-2.693980	-1.124727
36	6	3.917114	-2.980201	0.663494
37	1	2.965582	-0.429874	2.622414
38	6	4.036317	-2.247079	1.876533
39	1	4.522289	-3.836065	0.373312
40	1	4.748695	-2.446415	2.673830
41	26	4.362408	-1.012598	0.262468
42	6	4.799175	0.914368	-0.276850
43	1	4.157703	1.781445	-0.131864
44	6	5.770541	0.433450	0.646107
45	1	5.992905	0.856323	1.623350
46	6	6.388325	-0.713833	0.075335
47	1	7.160094	-1.321891	0.542058
48	6	4.818474	0.061457	-1.419932
49	1	4.195925	0.155658	-2.307422
50	6	5.801557	-0.944226	-1.199887
51	1	6.050349	-1.756914	-1.878784
52	6	1.296845	-0.313714	0.167457
53	1	1.456310	0.679044	0.607793
54	1	1.315849	-0.175290	-0.920827
55	7	-0.078301	2.670687	1.410693
56	6	0.275873	3.582870	2.027421
57	6	0.719004	4.723079	2.802669
58	1	0.245008	4.709505	3.793603
59	1	0.444255	5.654165	2.288382
60	1	1.810023	4.692061	2.926720
61	7	0.355499	2.207244	-1.979832
62	6	0.926542	2.820411	-2.776725
63	6	1.643917	3.586203	-3.775799
64	1	2.722793	3.552846	-3.571748
65	1	1.455608	3.169187	-4.774436
66	1	1.309215	4.632315	-3.757885

Table S10. Optimized geometry calculated for $[Pb(\mathbf{L3})(CH_3CN)_3]^{2+}$ at DFT level (mPW1PW/Def2SVP) in the gas phase in orthogonal Cartesian coordinate format.

Center Number	Atomic Number	Coordinates (Angstroms) X	Y	Z
1	82	-1.507751	0.788812	0.294535
2	16	-2.050724	-0.919426	2.637448
3	7	-3.229783	-1.311072	-0.261463
4	6	-3.816335	-2.011911	0.720566
5	16	-1.186013	-0.145003	-2.422911
6	7	0.322831	-1.113578	0.290576
7	6	-4.593243	-3.143150	0.459552
8	1	-5.047647	-3.695781	1.282451
9	6	-4.774170	-3.550571	-0.856319
10	1	-5.370887	-4.434001	-1.088687
11	6	-4.172779	-2.814256	-1.869387
12	1	-4.291831	-3.102788	-2.914184
13	6	-3.409898	-1.693154	-1.534147
14	6	-2.832541	-0.863854	-2.650405
15	1	-3.467370	0.022383	-2.812676
16	1	-2.831552	-1.432880	-3.589438
17	6	-0.088800	-1.575842	-2.165165
18	1	-0.414453	-2.380931	-2.839180
19	1	0.881632	-1.233198	-2.546273
20	6	0.021356	-2.114083	-0.746942
21	1	-0.914435	-2.617163	-0.476324
22	1	0.811581	-2.887013	-0.756240
23	6	0.390209	-1.760764	1.617266
24	1	0.817474	-1.028626	2.316858
25	1	1.100541	-2.604609	1.580263
26	6	-0.919930	-2.281604	2.182904
27	1	-0.702434	-2.853309	3.095565
28	1	-1.441461	-2.958738	1.493310
29	6	-3.674684	-1.554572	2.149505
30	1	-3.964538	-2.360540	2.836288
31	1	-4.361007	-0.715415	2.349530
32	6	2.840458	-1.247693	-0.002997
33	6	3.418840	-1.896112	-1.147403
34	6	3.667833	-1.570404	1.125655
35	1	3.068767	-1.832817	-2.176364
36	6	4.578516	-2.600965	-0.722121
37	1	3.528104	-1.215117	2.145112
38	6	4.731629	-2.400121	0.677643
39	1	5.250175	-3.167903	-1.362738
40	1	5.541047	-2.787621	1.292134
41	26	4.737533	-0.593442	-0.306428
42	6	4.868114	1.430228	-0.028108
43	1	4.139867	2.050493	0.490922
44	6	5.986024	0.781850	0.569719
45	1	6.256452	0.809052	1.622979
46	6	6.678325	0.070747	-0.449609
47	1	7.565835	-0.542405	-0.309921
48	6	4.871835	1.118202	-1.420076
49	1	4.148580	1.454324	-2.160446
50	6	5.991687	0.277730	-1.678205
51	1	6.265959	-0.146821	-2.641401
52	6	1.612343	-0.392422	0.027077
53	1	1.704035	0.369042	0.812894
54	1	1.503125	0.154994	-0.918161
55	7	0.111281	1.715210	2.489217
56	6	0.443689	2.310333	3.423565
57	6	0.860544	3.052815	4.596425
58	1	0.415781	2.610765	5.498233
59	1	0.534574	4.098461	4.514214
60	1	1.954715	3.027253	4.688794
61	7	0.249495	2.672483	-0.914944
62	7	-3.823335	2.055782	-0.945495
63	6	-4.678731	2.797259	-1.182837
64	6	-5.751686	3.724503	-1.483073
65	1	-6.716895	3.200515	-1.470219
66	1	-5.773111	4.528380	-0.734899
67	1	-5.598889	4.166773	-2.476791
68	6	0.798412	3.572762	-1.388615
69	6	1.491232	4.698459	-1.983635
70	1	1.257643	4.759732	-3.055096
71	1	1.178933	5.632140	-1.496802

72	1	2.576341	4.578945	-1.861257

Table S11. Optimized geometry calculated for [Pb(**L3**)(CH_3CN)$_4$]$^{2+}$ at DFT level (mPW1PW/Def2SVP) in the gas phase in orthogonal Cartesian coordinate format.

Center Number	Atomic Number	Coordinates (Angstroms) X	Y	Z
1	82	-1.422080	0.555851	0.002997
2	16	-1.902264	-0.739756	2.586571
3	7	-2.861950	-1.792743	-0.232152
4	6	-3.441672	-2.336054	0.848984
5	16	-0.742610	-0.917375	-2.453898
6	7	0.621572	-1.200859	0.480623
7	6	-4.061889	-3.586743	0.804649
8	1	-4.515288	-4.003637	1.704359
9	6	-4.087593	-4.286680	-0.395518
10	1	-4.560476	-5.268015	-0.457720
11	6	-3.488528	-3.716399	-1.511350
12	1	-3.484045	-4.237109	-2.469342
13	6	-2.885859	-2.461064	-1.394012
14	6	-2.296107	-1.830856	-2.627305
15	1	-2.989374	-1.067564	-3.014476
16	1	-2.171467	-2.583559	-3.416919
17	6	0.440015	-2.163424	-1.853157
18	1	0.234022	-3.111414	-2.370367
19	1	1.407624	-1.813675	-2.235187
20	6	0.483053	-2.404195	-0.351929
21	1	-0.429696	-2.929390	-0.045247
22	1	1.326461	-3.093970	-0.160168
23	6	0.646216	-1.571311	1.906351
24	1	0.968038	-0.685069	2.471886
25	1	1.414806	-2.344556	2.082945
26	6	-0.664727	-2.077139	2.487093
27	1	-0.480000	-2.444994	3.505686
28	1	-1.086162	-2.912492	1.912010
29	6	-3.451469	-1.568074	2.143236
30	1	-3.763950	-2.218581	2.970152
31	1	-4.175103	-0.739772	2.086992
32	6	3.155142	-1.112409	0.366268
33	6	3.873065	-1.930622	-0.570440
34	6	3.930857	-1.078034	1.573417
35	1	3.588740	-2.141264	-1.599924
36	6	5.066947	-2.384719	0.055416
37	1	3.684296	-0.522164	2.476323
38	6	5.102548	-1.858813	1.376501
39	1	5.834643	-3.000654	-0.407417
40	1	5.902546	-2.003877	2.098907
41	26	4.994347	-0.331111	0.003550
42	6	4.889108	1.702480	-0.193484
43	1	4.062060	2.329430	0.134449
44	6	6.025049	1.346041	0.588177
45	1	6.216248	1.644176	1.616659
46	6	6.859434	0.510005	-0.204932
47	1	7.795060	0.055724	0.113390
48	6	5.024506	1.084625	-1.472364
49	1	4.322128	1.151037	-2.300981
50	6	6.243110	0.347994	-1.476875
51	1	6.627962	-0.248908	-2.300741
52	6	1.847050	-0.414386	0.147057
53	1	1.806951	0.496430	0.759066
54	1	1.761199	-0.082512	-0.895841
55	7	0.052561	2.033356	2.014487
56	6	0.389427	2.797838	2.813636
57	6	0.814809	3.753459	3.817792
58	1	0.375520	3.497246	4.791241
59	1	0.492390	4.764646	3.535559
60	1	1.909366	3.742633	3.907923
61	7	0.362389	2.333371	-1.467545
62	7	-3.256121	1.381328	-2.148670
63	6	-3.910118	1.994954	-2.878325
64	6	-4.731711	2.759583	-3.796827
65	1	-5.770414	2.404833	-3.758302
66	1	-4.708535	3.823700	-3.526368

67	1	-4.355385	2.646065	-4.822398
68	6	0.964603	3.113468	-2.071411
69	6	1.726093	4.088433	-2.828629
70	1	1.589709	3.919397	-3.905180
71	1	1.388970	5.104214	-2.582483
72	1	2.794230	4.000650	-2.587572
73	7	-3.788912	1.755254	1.346272
74	6	-4.586547	2.460993	1.796400
75	6	-5.590104	3.343038	2.360722
76	1	-5.572834	3.281109	3.457139
77	1	-5.391437	4.380550	2.060387
78	1	-6.588292	3.054645	2.004774

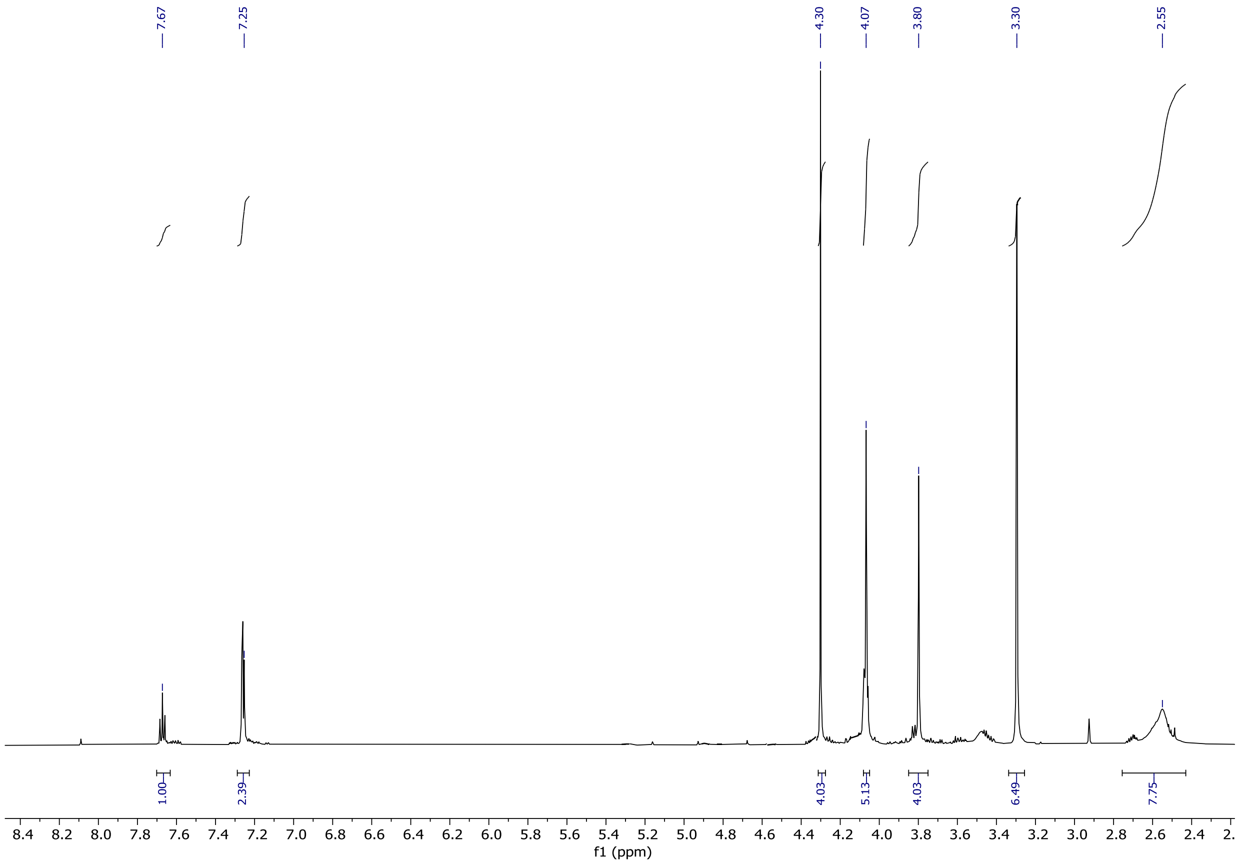

Figure S3. ^{1}H-NMR (600 MHz, $CDCl_3$) of **L3**.

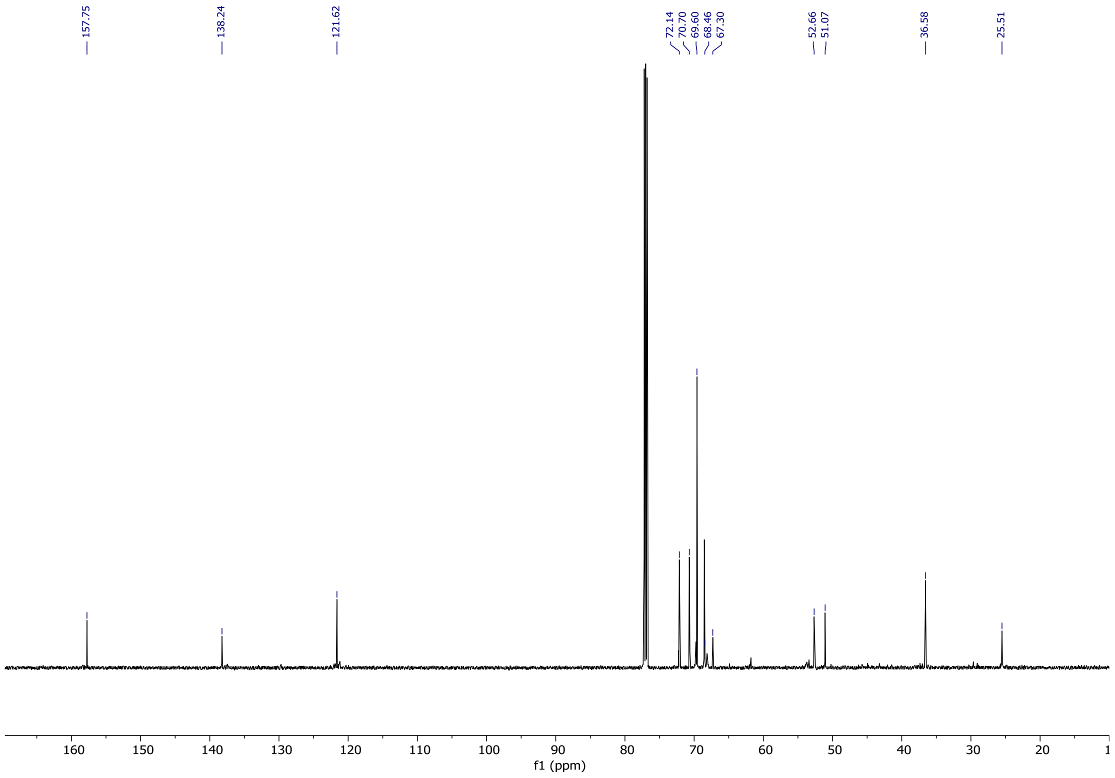

Figure S4. ^{13}C-NMR (150 MHz, $CDCl_3$) of **L3**.

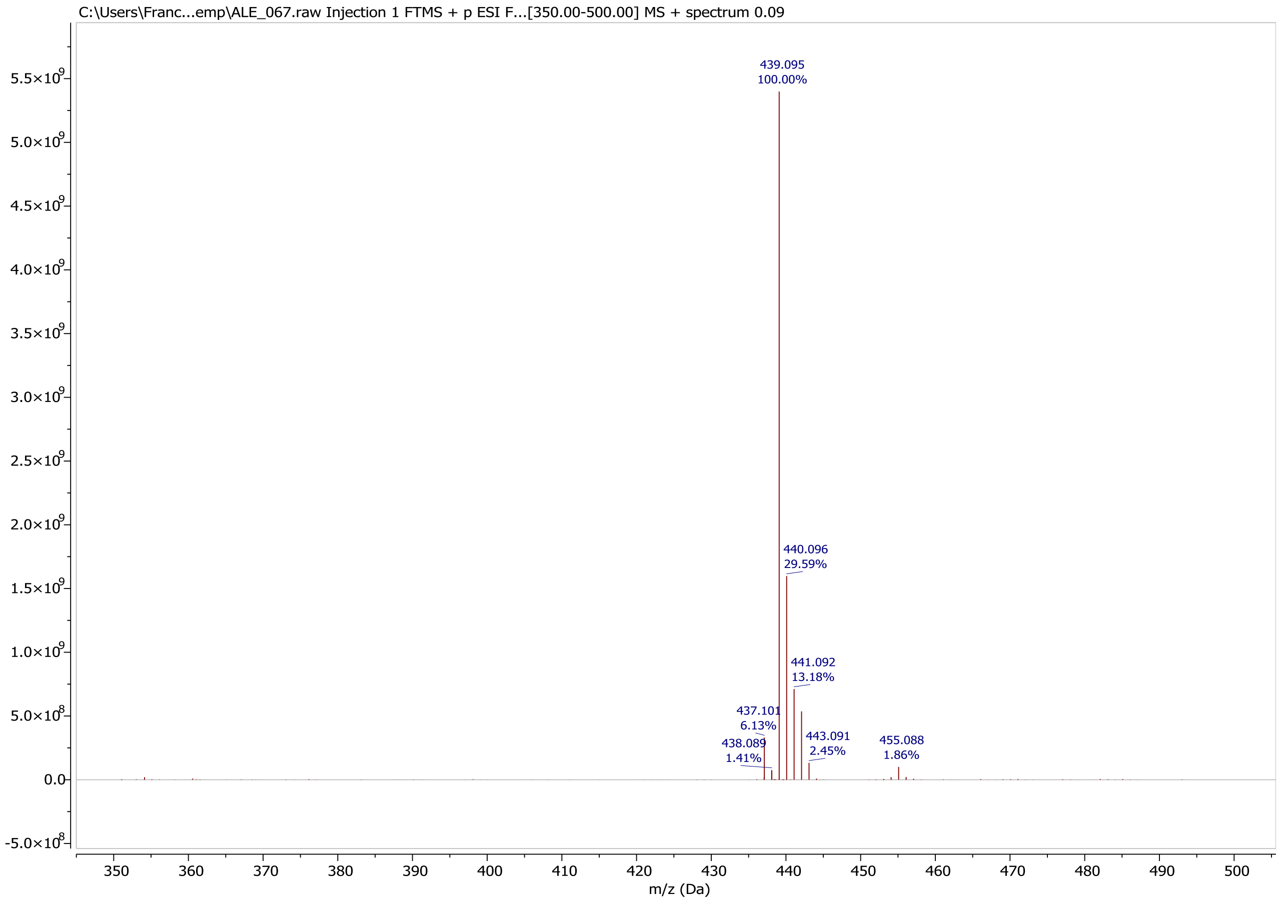

Figure S5. ESI(+) MS (CH_3CN solution) of **L3**.
